# Regenerable Cu-intercalated MnO_2_ layered cathode for highly cyclable energy dense batteries

**DOI:** 10.1038/ncomms14424

**Published:** 2017-03-06

**Authors:** Gautam G. Yadav, Joshua W. Gallaway, Damon E. Turney, Michael Nyce, Jinchao Huang, Xia Wei, Sanjoy Banerjee

**Affiliations:** 1 The CUNY Energy Institute at the City College of New York, Department of Chemical Engineering, Steinman Hall, 140th Street and 160 Convent Avenue, Room 316, New York, New York 10031, USA

## Abstract

Manganese dioxide cathodes are inexpensive and have high theoretical capacity (based on two electrons) of 617 mAh g^−1^, making them attractive for low-cost, energy-dense batteries. They are used in non-rechargeable batteries with anodes like zinc. Only ∼10% of the theoretical capacity is currently accessible in rechargeable alkaline systems. Attempts to access the full capacity using additives have been unsuccessful. We report a class of Bi-birnessite (a layered manganese oxide polymorph mixed with bismuth oxide (Bi_2_O_3_)) cathodes intercalated with Cu^2+^ that deliver near-full two-electron capacity reversibly for >6,000 cycles. The key to rechargeability lies in exploiting the redox potentials of Cu to reversibly intercalate into the Bi-birnessite-layered structure during its dissolution and precipitation process for stabilizing and enhancing its charge transfer characteristics. This process holds promise for other applications like catalysis and intercalation of metal ions into layered structures. A large prismatic rechargeable Zn-birnessite cell delivering ∼140 Wh l^−1^ is shown.

## Abstract (web summary)

Manganese oxide cathodes in alkaline solutions combine low cost and high capacity for energy storage, but it has been challenging to combine high capacity and stable cycling in this system. Here authors demonstrate reversible, high-capacity cycling when copper additives are introduced and investigate the transformations involved.

Batteries for grid applications such as integration of renewable power must be low cost, of high cycle life and energy density, safe, reliable and composed of easily acquired materials requiring relatively simple manufacturing processes^1^. Available technologies for grid applications are often unsuitable for wide deployment because of cost, durability and potential safety hazards^2^,^3^,^4^. High battery energy density is also desirable to minimize installation footprint, for example, for siting in urban areas. Manganese oxide (MnO_2_) has the desired attributes as an electrode material, being abundant, non-flammable, non-toxic, inexpensive, water-compatible and with a high gravimetric capacity of 617 mAh( g—MnO_2_)^−1^ (refs 5, 6, 7, 8, 9, 10, 11, 12, 13, 14, 15, 16, 17, 18, 19, 20, 21, 22, 23, 24). Commonly used primary MnO_2_ batteries, where electrolytic manganese dioxide (EMD or γ-MnO_2_) is paired with zinc (Zn) anodes, have very high-energy densities, >400 Wh l^−1^, but can only be discharged once owing to irreversible changes in the γ-MnO_2_ crystal structure^13^,^18^,^19^,^20^,^21^,^22^. Limiting the depth of discharge (DOD) to 5–10% of the 617 mAh g^−1^ MnO_2_ gravimetric capacity preserves the reversibility for 1,000–3,000 cycles but reduces energy density to 20 Wh l^−1^ (ref. 22). The low cost of the raw materials makes such low DOD MnO_2_-Zn batteries attractive for grid storage, with costs and cycle life close to the ARPA-E set targets (http://arpa-e.energy.gov/sites/default/files/documents/files/Volume%201_ARPA-E_ImpactSheetCompilation_FINAL.pdf). Nonetheless, access of the full second electron capacity of MnO_2_ with high cycle life would enable dramatically increased energy density and reduced costs for MnO_2_-Zn batteries.

High-energy density batteries for ‘real-world' applications require electrodes with a combination of high weight percent (wt%) loading of active materials and high areal capacity (mAh cm^−2^), which together would result in high energy density. Unfortunately high wt% loading is often the only parameter reported, which is not the true representation of an energy-dense battery as electrodes can be impractically thin with low active mass per unit area. Among the various polymorphs of manganese dioxide, the birnessite-phase (δ-MnO_2_) has been known to deliver ∼60–80% of the 617 mAh g^−1^ when cycled at low active mass loadings and under potentiodynamic protocols^15^,^16^,^17^. The rechargeability of the δ-MnO_2_ in these prior reports was achieved through additives like Bi_2_O_3_, which mitigated the effects of hausmannite (Mn_3_O_4_), an electrochemical-inactive phase^15^,^16^,^17^, through [Bi-Mn] complex interactions that maintained the layered structure of birnessite^23^,^24^. However, capacity fade was still an issue, and under galvanostatic cycling protocols, the additives had minimal effect, especially when the wt% loadings or areal capacity of the δ-MnO_2_ were high^25^,^26^. The addition of Bi_2_O_3_ was useful for low loadings of MnO_2_; however, at high loadings and galvanostatic cycling, the conductivity of the electrode is crucial, where fast charge transfer characteristics are required^27^. δ-MnO_2_ is a very resistive material^28^, and at high loadings, its poor charge transfer characteristics tend to result in the formation of Mn_3_O_4_ (ref. 27).

Prior reports have reported intercalating the δ-MnO_2_-layered structure with ions like cobalt (Co^2+^, Pb^2+^, Ni^2+^) to improve the electrochemical characteristics for Li-ion batteries^29^,^30^,^31^. However, these intercalants are toxic and/or expensive. In other fields, Cu has been used as an intercalant to improve the properties of layered structures like bismuth selenide^32^,^33^. Cu as an intercalant is attractive in cost and is non-toxic compared to the aforementioned intercalants and it has been shown to improve the electrochemical properties of birnessite^34^,^35^.

Here we report the development of Cu^2+^-intercalated layered MnO_2_ cathodes with a combination of high wt% loading and high areal capacity (mAh cm^−2^). These cathodes exhibit high volumetric capacity (mAh ml^−1^) and can be regenerated for several thousand charge–discharge cycles delivering nearly the two-electron capacity with minimal capacity fade and at high rate. Cu^2+^ intercalated Bi-birnessite (Bi-δ-MnO_2_) is a layered polymorph of MnO_2_ mixed with Bi_2_O_3_ and it regenerates via dissolution–precipitation during charge and discharge. The material exploits the redox potential of Cu to intercalate Cu^2+^ within the interlayer regions of Bi-δ-MnO_2_ during charging, and reduces it as Cu^0^ with a Mn(OH)_2_-layered material during discharge. As a consequence, the electrode charge transfer characteristics are greatly improved. This holds promise of dramatically improving energy density and energy storage cost and safety, and amplifies recent interest in birnessite for design of catalysts^36^, lithium (Li) intercalation materials^37^,^38^,^39^ and intercalation of metallic ions into layered structures^32^. A large-format rechargeable battery with high loadings of Cu^2+^-intercalated Bi-δ-MnO_2_ paired with Zn anodes delivering ∼140 Wh l^−1^ is shown.

## Results

### Electrochemical performance of Bi-δ-MnO_2_ with and without Cu

Birnessite-layered structure is of resurgent interest for battery applications as an ionic intercalation material^37^,^38^,^39^ and with what appears to be some characteristics of a conversion electrode^14^,^15^,^16^,^17^,^27^. Bi-δ-MnO_2_ in alkaline electrolyte is synthesized either by an *in-situ* formation step^15^,^16^,^26^,^40^,^41^ by discharging a mix of EMD with Bi_2_O_3_ and recharging to its charged state to form Bi-δ-MnO_2_ (see Methods section) or by *ex situ* synthesis methods^14^,^15^,^17^,^23^,^26^,^41^,^42^,^43^,^44^. Both methods have been shown to deliver 60–80% of δ-MnO_2_'s theoretical capacity (617 mAh g^−1^) at low areal loadings for hundreds of cycles potentiodynamically albeit with constant fade over cycle life^15^,^16^,^17^. Galvanostatically it has been cyclable at low mass loadings with reduced capacity retention^15^,^16^,^17^,^26^. The best results with higher wt% loading from a Bi-δ-MnO_2_ cathode were from Kannan *et al*.,^42^ where they showed an *ex situ* synthesized material could retain ∼310–500 mAh g^−1^ for ∼200–600 cycles at C/2 with 50–65 wt% loading. However, there was no mention of areal capacity in the paper and a recent report^44^ that used the same synthesis procedure to make Bi-δ-MnO_2_ found that at high areal capacity the cycle life was limited to 60 cycles. To the best of our knowledge, there has been no literature data accessing 80–95% of the 617 mAh g^−1^ of MnO_2_ at high mass loadings and areal capacities for thousands of cycles at C rates of interest.

**Figure 1:**
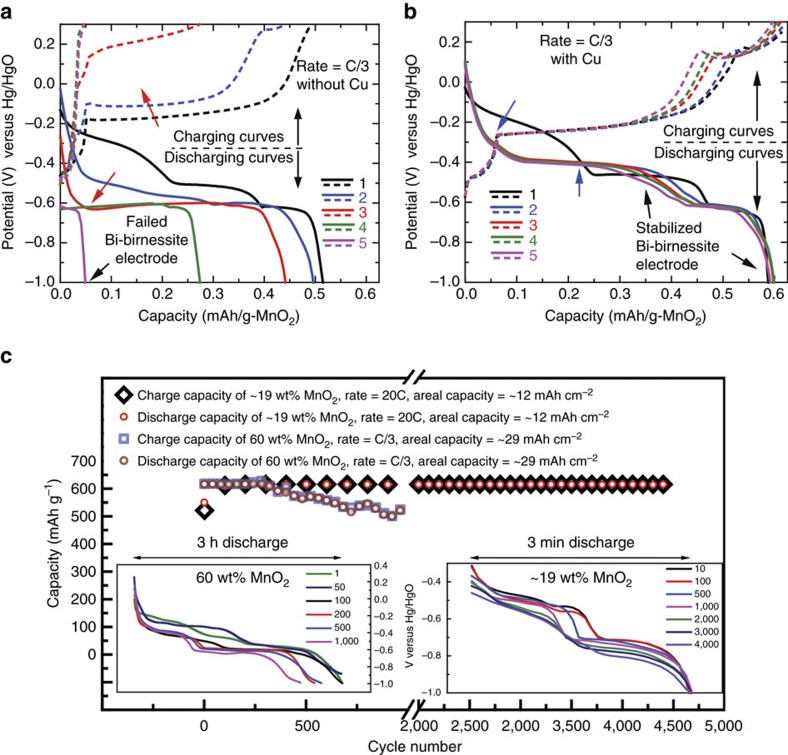
Galvanostatic data for Bi-birnessite cathodes with and without Cu additive. (**a**) First five C/3 cycles of a 45 wt% (∼21 mAh cm^−2^) EMD, Bi_2_O_3_ and carbon containing cathode fades completely in five cycles. During the first discharge, potentials characteristic of γ-MnO_2_ were seen. In the second cycle, potentials characteristic of a δ-MnO_2_ layered phase were seen. (**b**) A comparable electrode with Cu shows negligible capacity fade in five C/3 cycles. (**c**) Capacity versus cycle number for ∼19 and 60 wt% EMD, Bi_2_O_3_, Cu and carbon containing cathode at 20C and C/3, respectively. Areal capacities of the ∼19 and 60 wt% EMD cells were ∼12 and 29 mAh cm^−2^, respectively, which are indicative of high active loading electrodes. All cycling was against NiOOH counter electrodes. The 60 wt% cell is still cycling at the time of this writing.

To indicate the effects of Cu intercalation on cathode performance, cycling data with and without Cu are presented in Fig. 1. Cycle one in Fig. 1a shows the material evolution and *in situ* formation (see Methods section) of Bi-δ-MnO_2_ with high areal capacity (21 mAh cm^−2^, 45 wt% loading). EMD is mixed with carbon and Bi_2_O_3_ and discharged completely to −1 V versus mercury/mercury oxide (Hg/HgO) at C/3 in 37 wt% potassium hydroxide (KOH) solution to form Mn(OH)_2_ with Bi. Mn(OH)_2_ with Bi is then charged up at C/3 to 0.3 V versus Hg/HgO to form Bi-δ-MnO_2_. Second cycle onwards the flat potential discharge characteristics of the δ-MnO_2_ cathode are seen^15^,^16^,^17^; however, it is not able to retain the capacity. The cause of failure appears to be due to Mn_3_O_4_ formation as discussed later in the manuscript.

Figure 1b shows the cycle performance of a Cu^2+^-intercalated Bi-δ-MnO_2_ cathode, which is able to access the theoretical capacity reversibly. The Cu^2+^-intercalated Bi-δ-MnO_2_ formation process is very similar, except with the addition of Cu to the mix of EMD, Bi_2_O_3_ and carbon. This mix is discharged completely to −1 V versus Hg/HgO and charged to 0.3 V versus Hg/HgO to form Cu^2+^-intercalated Bi-δ-MnO_2_. The effect of Cu is seen, and further cycling reveals that Cu addition is necessary for the stability of Bi-δ-MnO_2_. The discharge potentials (−0.406 V versus Hg/HgO) and charge potentials (−0.26 V versus Hg/HgO) for the Cu-containing electrode (blue arrows) were stable, while those of the electrode without Cu (red arrows) were not. Impedance experiments^45^ were performed at the end of charge (0.3 V versus Hg/HgO) for the first five cycles (Supplementary Fig. 1), showing the charge transfer resistance of the Cu-containing electrode to increase from 0.26 to 0.65 mΩ cm^−2^, whereas the resistance of the non-Cu electrode increased from 0.78 to 12.7 mΩ cm^−2^. High cycling rate (20C) and cycle life (>4,000) of the Cu-intercalated material with a loading of ∼19 wt% MnO_2_ (∼12 mAh cm^−2^) is demonstrated in Fig. 1c. The system in general has low charge transfer resistance and can operate at ultrahigh rates. A 60 wt% MnO_2_ loading (∼29 mAh cm^−2^) cell cycled at C/3 is also shown in Fig. 1c, where it is able to access the complete capacity for ∼300 cycles, after which it retains >80% of the 617 mAh g^−1^ over 1,000 cycles.

Further, for a series of cells, the wt% loading of EMD was varied from 5 to 45, and the cells were cycled galvanostatically from 1 C to 40 C against nickel oxyhydroxide (NiOOH) counter electrode (Supplementary Fig. 2). The cell performance was stable, delivering a large fraction of the two-electron theoretical capacity. Even at high cycling rates, a high loading cell (45 wt%,∼19 mAh cm^−2^) delivered ∼60–65% of the theoretical capacity in galvanostatic cycling at 4C for >1,700 cycles (Supplementary Fig. 2b). In several such experiments, much of the capacity was obtained for voltages above −0.6 V, where the effect of the Bi and Cu additives would be expected to contribute little as indicated by the potentiodynamic data in Supplementary Fig. 4. In some experiments, such as that shown in Supplementary Fig. 2c, the role of the additives on the capacity is more difficult to interpret because some portion of the discharge capacity is extracted at lower voltages. Nonetheless, potentiodynamic data indicate that even in this low voltage region a majority of the capacity is still derived from the MnO_2_ (Supplementary Figs 4 and 5). The potentials at which these capacities were obtained appeared to be dependent on wt% loadings and cycling rates. In practical applications, these conditions would need to be optimized. Statistical reliability of the data was also ensured by gathering the average discharge capacity of three similar cells with 50 wt% MnO_2_ cycled at 1C, which is shown in Supplementary Fig. 3, where the standard deviation of the discharged capacity is <2% of the mean.

### Regeneration mechanism and electrode characterization

**Figure 2:**
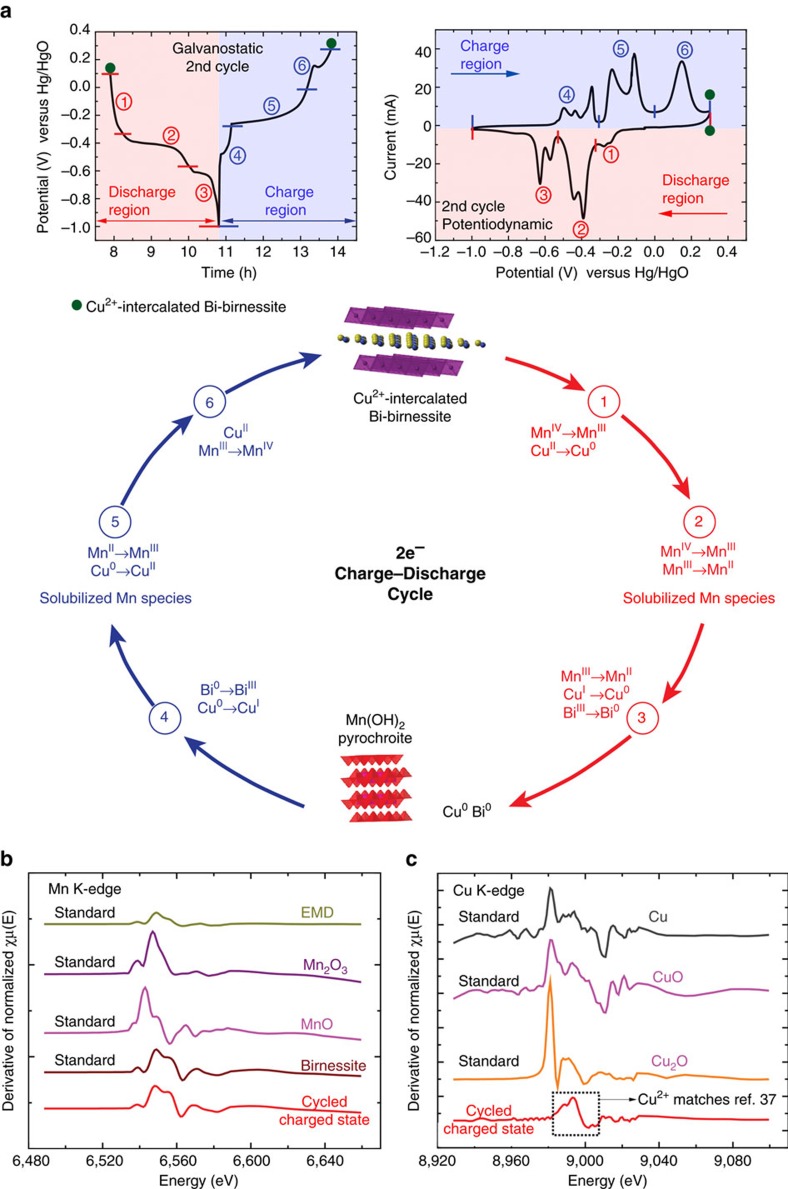
Regeneration mechanism and supporting XANES spectra. (**a**) Electrochemical reactions for the regeneration cycle of Cu^2+^-intercalated Bi-birnessite. The reactions taking place in each region are colour-coded and numbered corresponding to the galvanostatic and potentiodynamic curves. (**b**) XANES spectra of a cycled electrode in the charged state for the Mn K edge indicating match with birnessite and (**c**) for the Cu K-edge of the same material indicating that Cu exists as Cu^2+^ intercalated into birnessite matching ref. 37.

Figure 2a shows performance of the second cycle of a Cu^2+^-intercalated Bi-δ-MnO_2_ electrode under galvanostatic or potentiodynamic cycling. The slow potentiodynamic cycle (0.1 mV s^−1^) shows the potentials of electrochemical reactions from which the oxidation state of the reactions shown in Fig. 2a were derived. Detailed combinatorial cyclic voltammograms and the reactions are presented in Supplementary Figs 4–6 and Supplementary Table 1, where each component was methodically added and the electrode potentials were slowly swept to observe the faradaic reactions. The reaction mechanism of Cu^2+^-intercalated Bi-δ-MnO_2_ is illustrated in Fig. 2a, wherein three regions of reaction are observered during both charge and discharge. In the discharge region labelled 1, Mn^IV^ and Cu^II^ partially reduce to form Mn^III^ and Cu^0^. The partial reduction of Mn^IV^ is perhaps from surface sites. Discharge region 2 is where Bi-δ-MnO_2_ without Cu is previously known to undergo Mn^IV^ to Mn^III^ to Mn^II^ reactions, and here we find that Bi-δ-MnO_2_ with Cu also has its significant capacity. This reaction pathway includes a dissolved Mn^III^ state followed by formation of solid Mn(OH)_2_. In the discharge region 3, the remaining Cu^I^ is reduced to Cu^0^ and Bi^III^ to Bi^0^. These regions occur for short times and hence the capacity in those regions is small as supported by the low capacity below −0.6 V versus Hg/HgO in Fig. 1b. In the charge region labelled as 4, Bi^0^ oxidizes to Bi^III^ with partial oxidation of Cu^0^ to Cu^I^, then substantial charging occurs in region 5 where Mn^II^ is oxidized to Mn^III^. Just above the dissolution potential of Mn^II^, Cu^0^ also oxidizes to form Cu^II^, which is present as a dissolved species. In the charge region 6, the formation of Bi-δ-MnO_2_ (Mn^III^ to Mn^IV^) takes place, and Cu^II^ ions intercalate within the interlayer regions to completely reform Cu^2+^-intercalated Bi-δ-MnO_2_. The mechanism to the improved performance of the Cu^2+^-intercalated Bi-δ-MnO_2_ material is that Cu decreases the charge transfer resistance of the δ-MnO_2_, allowing complete regeneration of the materials from the dissolved state during each discharge and charge.

Reversible formation of Cu^2+^-intercalated Bi-δ-MnO_2_ is confirmed by XANES analysis of an electrode that was cycled 60 times and stopped on charge (Fig. 2b,c). The cycled electrode showed characteristic Mn K-edges of a birnessite phase, and also the Cu K-edge matches that of Cu^2+^ ions in birnessite^37^,^46^,^47^. The presence of Cu^2+^ ions reduces the oxidation state of Mn in Bi-birnessite as seen in Supplementary Fig. 7a.

**Figure 3:**
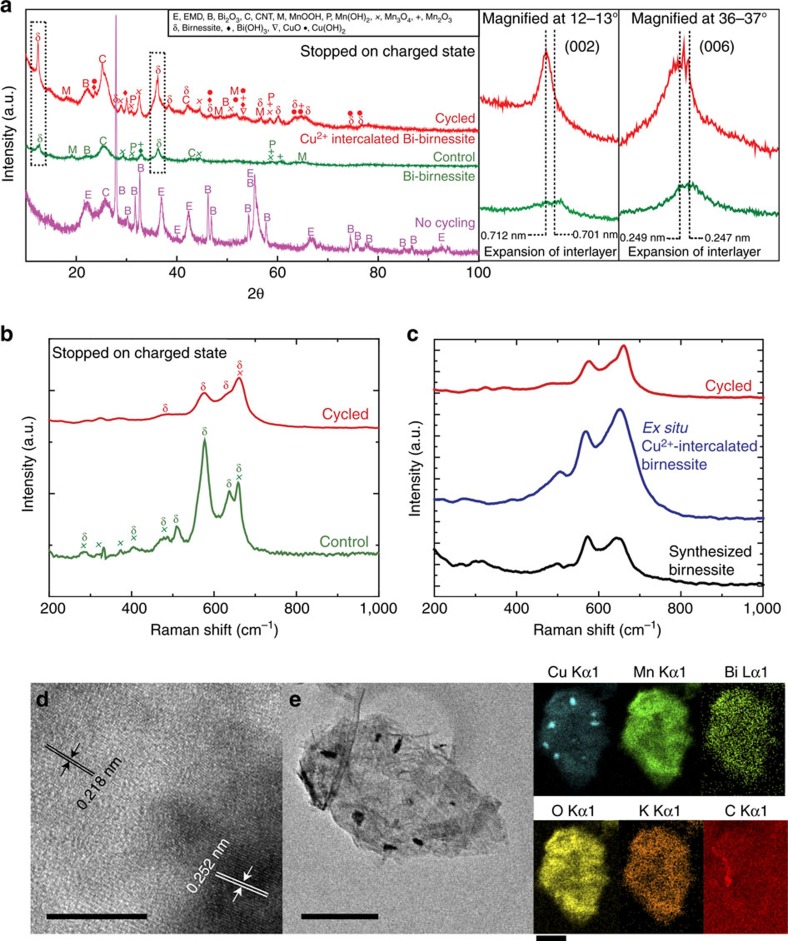
Characterization of cycled (with Cu) and control (no Cu) electrodes. (**a**) XRD of cycled Cu Bi-birnessite, cycled Bi-birnessite without Cu and uncycled initial electrode mix. Magnified panels show interlayer expansion in the case with Cu. (**b**) Raman spectra of cycled and control electrodes. (**c**) Raman spectra of cycled electrode, Cu^2+^-intercalated birnessite produced *ex situ* and synthetic birnessite. (**d**) HRTEM image of the cycled electrode. The darker region and lighter region is indexed to δ-MnO_2_. (**e**) TEM EDX mapping of a region of the cycled electrode. Scale bars in **d**,**e** are 10 and 500 nm, respectively. Scale bar for maps in **e** is 250 nm.

Figure 3 shows further characterization to confirm the intercalation of Cu^2+^ within the layers of birnessite on cycled electrodes (also see Supplementary Figs 7–11). Figure 3a shows a X-ray diffraction (XRD) comparison of three materials: a Cu Bi-birnessite electrode cycled 60 times, a control Bi-birnessite electrode with no Cu (failed after four cycles), and non-cycled initial electrode mix. The cycled and control (no-Cu) patterns are both composed predominantly of δ-MnO_2_ (indexed to hexagonal space group), indicating the successful Bi-δ-MnO_2_ formation process, distinct from the ‘no cyling' pattern. The c-direction peaks, corresponding to the birnessite interlayer spacing, are shown magnified on the right-side plot. With Cu present, the birnessite interlayer is expanded compared to the control electrode. This expansion is consistent with other reports where metal ions have been intercalated in the birnessite interlayers^31^,^48^. Other peaks are also seen in the XRD of the control electrode, which are indexed to Mn_3_O_4_. To further confirm the fingerprints of these respective phases, Raman spectra was taken for the control and cycled electrode at its charged state. The Raman spectra in Fig. 3b show peaks from the control material match to Mn_3_O_4_ (ref. 49), whereas different peaks dominate the cycled Cu Bi-δ-MnO_2_ material. Raman spectra for synthesized birnessites have the strongest intensity ∼575 cm^−1^ due to the Mn-O stretching along the chains in the MnO_6_ sheets followed by a band at ∼640–645 cm^−1^ due to the out of plane stretching of Mn-O in the MnO_6_ groups. However, in the Raman spectra of the cycled electrode a significant broadening feature of the band at ∼640–645 cm^−1^ is seen compared to the control electrode. This broadening feature could mean a disruption of the Mn-O caused by the presence of Cu^2+^ ions^50^. *Ex situ* synthetic birnessite was hydrothermally made^51^ and soaked in a Cu^2+^ solution (copper sulfate (CuSO_4_)) for 48 h, and Raman and XRD were performed. The XRD (Supplementary Fig. 7c) for the synthetic Cu-soaked birnessite showed the same peak shifts that were seen in the cycled Cu^2+^-intercalated Bi-birnessite material. The Raman spectra in Fig. 3c also show a match between synthetic birnessite soaked in Cu^2+^ and battery-cycled Cu^2+^ Bi-δ-MnO_2_, indicating expansion of the birnessite interlayer and pointing the Cu^2+^ ions as the origin of the broadening feature^36^,^50^,^52^. This shows that Cu^2+^ is intercalated in the Bi-δ-MnO_2_-layered structure during battery cycling, and that this structure is continually reformed upon charging the electrode.

Electron microscopy was also performed to understand the morphological effects of Cu^2+^ intercalation. The d-spacing expansion ((0 0 6) direction) from the high-resolution transmission electron microscopy image of the cycled electrode, as shown in Fig. 3d, corroborated the expansion seen in the XRD in Fig. 3a. The energy dispersive X-ray spectroscopy (EDX) mapping image in Fig. 3e and line scan (Supplementary Fig. 11) clearly show the colocation of Cu with Mn throughout the Bi-birnessite nanoparticle, while no Cu was seen in the electrode's surrounding carbon material. Scanning electron microscopy (SEM) and EDX analysis (Supplementary Fig. 8a,c) of the control electrode show an amorphous nature and a Mn to O ratio of ∼0.68, indicating Mn_3_O_4_ presence. SEM and EDX analysis of the cycled electrodes show sheet-type structures with Cu colocated with Mn (Supplementary Fig. 8b,d). EDX of the synthetic, Cu^2+^-soaked birnessite samples showed that Cu^2+^ had almost completely replaced the K^+^ ions present within the layers (Supplementary Fig. 9e–h).

### Large-format rechargeable energy-dense cell

**Figure 4:**
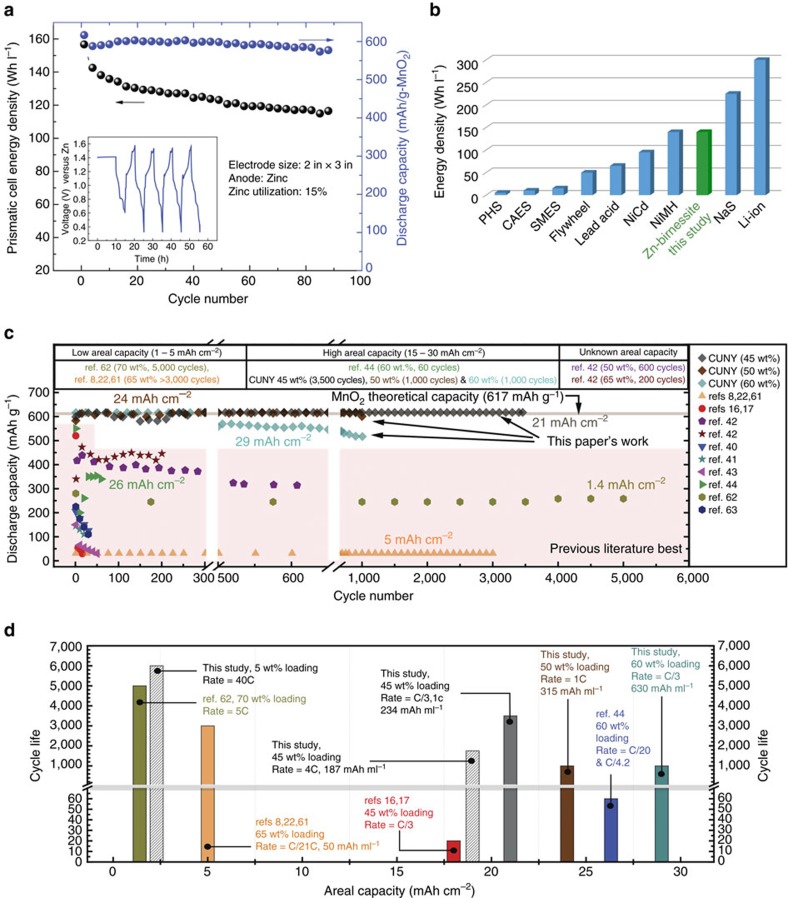
Performance characteristics of a Zn-birnessite cell. (**a**) The discharge energy density (Wh l^−1^) and capacity (mAh g^−1^) for a Zn-birnessite cell using the Cu^2+^-intercalated Bi-birnessite electrode with 60 wt% EMD in the initial mix (continues to cycle at the time of this writing). The voltage-time characteristics for the first 5 cycles are shown in the inset. The electrode sizes are 5.08 cm × 7.62 cm. (**b**) Energy density comparison of energy storage systems for grid applications^59^ indicating competitive performance of the Cu^2+^-intercalated Bi-birnessite. (**c**) Comparison of the best MnO_2_ cathodes reported in literature for aqueous Zn-MnO_2_ batteries. (**d**) Comparison of the areal capacities and cycle life obtained gathered from reported results in literature^60^,^61^,^62^,^63^. CAES, compressed air energy storage; PHS, pumped hydro storage; SMES, superconducting magnetic energy storage.

The *in situ* synthesis of the δ-MnO_2_ using inexpensive, abundant raw materials and the ease of manufacturability make the Cu^2+^-intercalated Bi-δ-MnO_2_ an attractive cathode. *Ex situ* synthesized δ-MnO_2_ can also be used with no difference in performance as shown in Supplementary Fig. 12. From a device standpoint, addition of Cu is not a significant cost contributor because low areal loadings of Cu are sufficient to result in the enhanced capacity and performance (Supplementary Fig. 13). This also indicates that the capacity obtained is predominantly associated with the δ-MnO_2_ as the effect of different Cu loadings on capacity is insignificant. We also cycled a Cu cathode against NiOOH counter electrode in a cell of similar design and found that the capacity was <5 mAh g^−1^ (Supplementary Fig. 14), which supported the finding that Cu added little to the overall capacity of the birnessite cells we tested. To demonstrate the practicality of a Cu^2+^-intercalated Bi-δ-MnO_2_ as a battery electrode, *in situ* synthesized materials were prefered as considerable time and money are saved in comparison to making *ex situ* synthesized birnessites. The Cu^2+^-intercalated Bi-δ-MnO_2_ electrode was cycled against a Zn anode in a large-format prismatic cell with industrial-strength loading of Mn per volume (60 wt%). The battery delivered an energy density of ∼160 Wh l^−1^ for the first cycle and then cycled ∼120 Wh l^−1^ for 90 cycles, as shown in Fig. 4a. This rechargeable aqueous battery possesses a high energy density relative to existing technology, as shown in Fig. 4b. Energy density is currently limited by the Zn DOD (15% in this cell). Zn anodes face cycle life challenges when cycled at more than 10% of theoretical capacity^53^. Calculation shows that when Cu^2+^-intercalated Bi-δ-MnO_2_ is paired with a Zn anode that cycles at ∼35% of its theoretical capacity, the resulting cell would deliver >250 Wh l^−1^, equal to some varieties of Li-ion battery. A comparison of the best MnO_2_ cathodes reported in literature is shown in Fig. 4c (see Supplementary Table 2), where a striking advancement in cycle life and capacity is noted for Cu^2+^-intercalated Bi-δ-MnO_2_ electrodes. An electrode with high areal capacity is preferred as more active mass is used meaning higher energy density and lower cost. Figure 4d illustrates the best areal capacity and cycle life from references in Fig. 4c. Obtaining high cycle life is easy when areal capacities are very low. We show for the first time the attainment of 80–100% of the second electron capacity of MnO_2_ with higher areal capacities for thousands of cycles, which also results in high volumetric capacity (mAh ml^−1^, see Supplementary Table 2).

In conclusion, an MnO_2_ cathode with an exceptional combination of reversibility and very high capacity is found to operate via Cu ions intercalated into birnessite-layered structures. The resulting composite material benefits from enhanced charge transfer and complete regeneration of layered materials on each cycle. The application of this methodology is not only limited to batteries but also applies to areas where layered materials are of interest like oxidation catalysts^36^, intercalation chemistry^37^,^38^,^39^ and membranes for removal of heavy-metal ions^54^.

## Methods

### Materials

Manganese sulfate, potassium permanganate and CuSO_4_ were purchased from Sigma-Aldrich. Electrolytic manganese dioxide (EMD or γ-MnO_2_, 92%) and bismuth oxide (Bi_2_O_3_, 99.8%) powders were purchased from Tronox Limited and Sigma-Aldrich, respectively. Carboxymethylcellulose sodium (CMC) and polyvinyl alcohol (PVA) were purchased from Sigma-Aldrich. An aqueous binder of 5 wt% CMC-PVA was made by repeated stirring and heating. NiOOH electrodes were purchased from Jiangsu Highstar Battery Manufacturing. Zn (>98%) and zinc oxide (>98%) powders were purchased from Umicore. Teflon was purchased from Dupont. Multiwalled carbon nanotubes (CNTs) were purchased from CNano Technology Limited. Graphite (KS44) was purchased from TIMCAL. KOH pellets were purchased from Fisher Scientific.

### Synthesis

A 6:1 molar solution of potassium permanganate and MnSO_4_ was taken in a beaker and mixed till the reactants were completely dissolved. The dissolved solution was kept in a Teflon container and heated at 160 °C for 12 h. The reactants were allowed to cool to room temperature and were finally washed with deionized water three times. The product that was formed was synthetic birnessite. For *ex situ* intercalation of Cu^2+^, 1 M solution of CuSO_4_ was created and mixed with the synthetic birnessite powder for 48 h. The powders were washed with deionized water three times and dried.

### Electrodes

The cathode mix was made by ball milling MnO_2_, Bi_2_O_3_ and CNT in appropriate proportions. Slurry was made by adding CMC-PVA and copper (metallic state) to the mix. The mix was pasted onto a nickel mesh, dried and pressed. For the cyclic voltammetry experiments, KS44 was used as the conductive additive. The NiOOH electrodes were commercially made and were used as received. The Zn anodes were made by wet mixing 85% Zn, 10% zinc oxide and 5% Teflon. The mix was then rolled into sheets with a certain thickness and dried at 60 °C for 2 h. The sheets were then cut into appropriate dimensions and pressed onto a copper mesh^55^,^56^. The utilization of the Zn was 15%.

### Cell assembly

The electrodes were assembled into a prismatic box (2.54 cm depth × 8.26 cm width × 21.6 cm height) made of polysulfone. The inner diameter of the polysulfone box where the electrodes are compressed is 1.94 cm. Cellophane was used as the separator in the cells that contained NiOOH as the counter electrode. The NiOOH electrodes were sealed in polyolefin nonwoven membrane (FS 2192SG; Freudenberg). The cathodes were wrapped with three layers of cellophane and then paired with two NiOOH electrodes and uniformly compressed in the prismatic box with polypropylene shims. The electrode sizes tested in this cell assembly were 2.54 cm × 2.54 cm and 5.08 cm × 7.62 cm, respectively. In the Zn-birnessite cell, the electrodes were fitted in the above polysulfone box as well. The cathode was wrapped in three layers of cellophane, and the Zn anode was sealed in polyolefin nonwoven membrane (FS 2192SG; Freudenberg) and Celgard 5550 (Celgard). A pack consists of one cathode and anode with the aforementioned separators was compressed in the prismatic box with polypropylene shims. The pack's total thickness of 0.163 cm consists of 0.046 cm cathode, 0.058 cm anode, 0.013 cm cellophane, 0.030 cm FS 2192SG and 0.015 cm Celgard 5550. A volume of 0.0063 L is calculated from the electrode area (5.08 cm × 7.62 cm) times the pack thickness.

### Electrochemical tests

The impedance experiments (EIS) were performed on 5.08 cm × 7.62 cm electrodes. NiOOH electrodes were used as the counter electrode. The cathodes used in the tests had 45 wt% with the balance being covered by Bi_2_O_3_, CNT and CMC-PVA for the Bi-birnessite electrode, and the same mix with Cu for the Cu^2+^-intercalated Bi-birnessite. The EIS was performed at a frequency range of 20 kHz to 0.1 Hz at an AC amplitude of 5 mV. The cyclic voltammetry experiments were performed on a 2.54 cm × 2.54 cm electrode. NiOOH electrodes were used as the counter electrode. The cathodes used in the tests had 5 wt% active material loading of MnO_2_ with the remaining balance covered by Bi_2_O_3_, Cu, KS44 and CMC-PVA. In the tests where there was no MnO_2_, the main active material was Bi_2_O_3_. The weight ratio of KS-44 to active material was ∼17. 37 wt%. KOH was used as the electrolyte. A Hg/HgO reference electrode was used to monitor the cathode. The tests were run at 0.1 mV s^−1^ between 0.3 and −1 V against the reference. The tests were conducted on a Biologic potentiostat/galvanostat (VSP Modular 5-channel) instrument. The galvanostatic experiments were performed on a 2.54 cm × 2.54 cm and 5.08 cm × 7.62 cm electrodes. NiOOH or Zn was used as the counter electrodes. The cathode had 45–60 wt% loading of MnO_2_ with the balance being covered by Bi_2_O_3_, Cu, CNT and CMC-PVA. The 60 wt% MnO_2_ loading electrode did not contain any CMC-PVA. The molar ratio of Bi_2_O_3_ to MnO_2_ was around 0.04 irrespective of MnO_2_ loading percentages. For the 10 C and 20 C tests, around 19 wt% loading of MnO_2_ was used, and for the 40 C test, around 5 wt% MnO_2_ loading was used. KOH of 25 wt% was used for the Zn anodes and 37 wt% of KOH was used for the NiOOH counter electrodes. A Hg/HgO reference electrode was used in the tests where NiOOH was the counter electrode. The tests were run at C/3 against Zn, and at C/3, 1 C, 4 C, 10 C, 20 C and 40 C in the cells with NiOOH as the counter electrode. All the capacity calculations are based on the mass of MnO_2_ used in the mix. The cell cycling protocols were set to access the 617 mAh g^−1^ (theoretical limit of MnO_2_) between 0.3 and −1 V versus Hg/HgO. The galvanostatic tests were carried out on a multichannel Arbin BT 2000.

### *In situ* synthesis of Bi-birnessite

Cycle one (black curves) in Fig. 1a shows the electrochemical *in situ* formation process of Bi-δ-MnO_2_ against a NiOOH counter electrode. EMD (45 wt%) is mixed with carbon powder and Bi_2_O_3_ and discharged completely (to −1 V versus Hg/HgO, black solid curve) at C/3 in 37 wt% KOH solution to form Mn(OH)_2_. The sigmoidal part (∼−0.15 to −0.5 V versus Hg/HgO) of the solid black curve indicates the proton (H^+^) intercalation process into the tunnel structure of EMD to form MnOOH. This represents the first electron reaction, which delivers ∼308 mAh g^−1^.


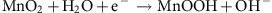


The flat part of the solid black curves (∼−0.5 to −1 V versus Hg/HgO) represents the second electron reaction (delivering ∼308 mAh g^−1^), where the dissolution–precipitation of the Mn ions^57^,^58^ from MnOOH to form Mn(OH)_2_ and Bi_2_O_3_ reduction to form Bi metal (see Supplementary Table 1) takes place.


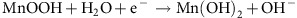


The discharged product (Mn(OH)_2_ with Bi) is then charged up at C/3 to birnessite's formation potentials (0.3 V versus Hg/HgO; Supplementary Table 1 has detailed reactions) to finally form Bi-δ-MnO_2_. The Bi_2_O_3_ role in this process is to maintain the layered structure through [Bi-Mn] interactions^32^,^33^, which are seen in the peaks of potentiodynamic cycling (Fig. 2) and to a lesser extent in the galvanostatic curves of Fig. 1a. Second cycle onwards the flat potential discharge characteristics (solid blue, red, green and pink curves) of the Bi-δ-MnO_2_ cathode are seen. The Cu^2+^ intercalated Bi-δ-MnO_2_ is formed through the same *in situ* formation step, except with the addition of Cu to the mix of EMD, Bi_2_O_3_ and carbon.

### Material characterization

XRD and Raman spectroscopy were performed on all the materials. The materials were also viewed under an SEM and a TEM. XRD was carried out on a PANalytical X'Pert Pro Powder Diffraction instrument fitted with a PIXcel^1D^ fast detector and Cu Kα filter. Raman measurements were taken on a MonoVista Confocal Raman Microscope with SP 2750 series spectrograph from Princeton Instruments-Action and an upright Olympus BX51 confocal microscope. SEM images were taken on a Zeiss Supra 55 VP field emission microscopy fitted with an EDX. TEM images were taken on a JEOL JEM2100 microscope fitted with an Inca EDX system, and also with STEM (scanning transmission electron microscopy) capabilities. Gold grids were used for the TEM and EDX analyses. XANES was taken on the 5-ID (SRX) beamline of the National Synchrotron Light Source II at Brookhaven National Laboratory, New York. Multiple measurements were taken for different MnO_2_ and copper standards. For the Mn K-edge, EMD, MnO, Mn_2_O_3_ and synthesized birnessite were used for the Mn standards. For the Cu K-edge, metallic Cu, CuO and Cu_2_O were used for the Cu standards. All the XANES data was analysed in Athena. The characterization work was performed on *ex situ* samples, which were washed and dried in their charged states, which could have resulted in some oxidation, perhaps explaining the minor peak of CuO in the XRD.

### Data availability

The authors declare that the data supporting the findings of this study are available from the corresponding author on reasonable request.

## Additional information

**How to cite this article:** Yadav, G. G. *et al*. Regenerable Cu-intercalated MnO_2_ layered cathode for highly cyclable energy dense batteries. *Nat. Commun.* **8,** 14424 doi: 10.1038/ncomms14424 (2017).

**Publisher's note**: Springer Nature remains neutral with regard to jurisdictional claims in published maps and institutional affiliations.

## Supplementary Material

Supplementary Information Supplementary Figures, Supplementary Tables, Supplementary Discussion and Supplementary References
