# Supplementary material for: Regenerable Cu-intercalated MnO2 layered cathode for highly cyclable energy dense batteries
Source: Nat Commun. 2017 Mar 6;8:14424. doi: 10.1038/ncomms14424 (PMC5343464; doi:10.1038/ncomms14424)
Supplement: Supplementary Information — Supplementary Figures, Supplementary Tables, Supplementary Discussion and Supplementary References [file ncomms14424-s1.pdf]

Supplementary Information

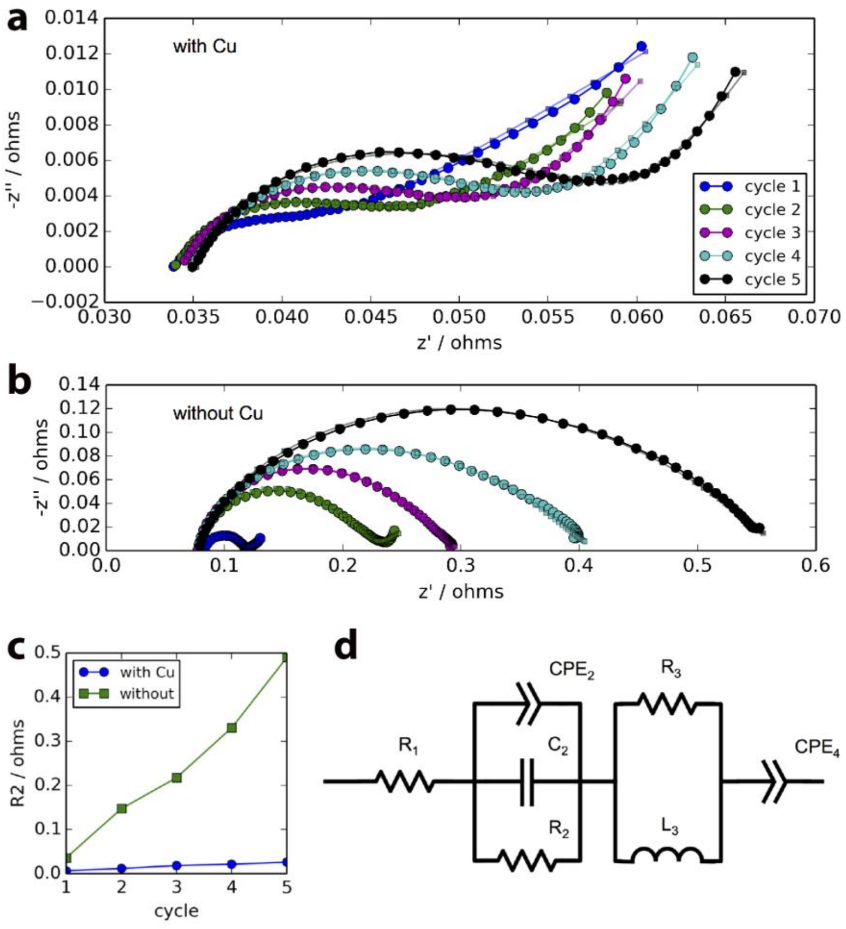

Supplementary Figure 1| Impedance analysis at the end of charge for the first five cycles for Cu^{2+}intercalated Bi-birnessite and Bi-birnessite. a, Cell impedance for the first five cycles in Cu^{2+} intercalated Bi-birnessite cell. The transparent lines represent the fit. **b,** Cell impedance for the first five cycles in Bi-birnessite cell. The transparent lines represent the fit. **c,** Comparison of charge transfer resistances in Cu^{2+} intercalated Bi-birnessite and Bi-birnessite cells. **d,** Equivalent circuit model that was used to fit the impedance curves. This model was based on the MnO_2 equivalent circuit model developed by reference 46. R_1 is the solution resistance; R_2 is the resistance to charge transfer at the cathode-electrolyte interface, CPE_2 is the constant phase element for nonfaradaic charging of the double layer, C_2 is the capacitance of the carbon support material; L_3 and R_3 are the inductance and resistance associated with OH- transport in the interlayer of δ-MnO_2; and CPE_4 accounts for semi-infinite diffusion to the electrode surface, which has a significant surface roughness. R_2, which is a measure of electrode kinetics, captures two phenomena: 1) the charge transfer coefficient of the electrochemical reaction and 2) any change in the active interfacial area caused by better electron percolation within the porous electrode.

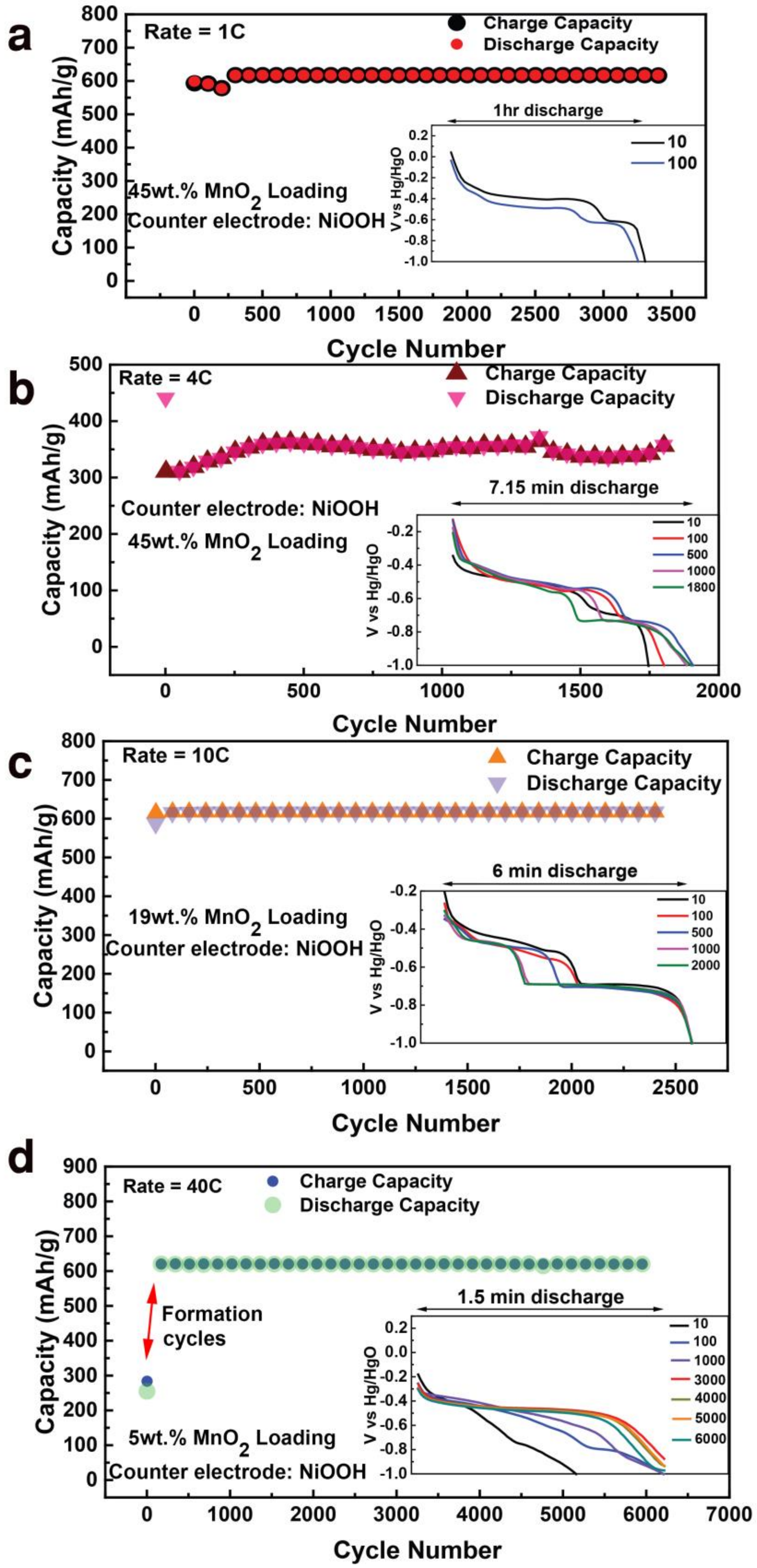

Supplementary Figure 2| Rate characteristics of the Cu^{2+} intercalated Bi-birnessite. **a,** Capacity(mAh/g) vs cycle number for the Cu^{2+} intercalated Bi-birnessite electrode is

shown. The cell is cycled at 1C charge and discharge rate. The voltage-time curves for the different cycles is shown in the inset. **b,** Capacity versus cycle number for 45wt.% MnO_2 loading electrode ran at 4C is shown. A high capacity of ~380mAh/g after ~1800 cycles is obtained with near 100% coulombic efficiency. Inset shows the discharge curves for various cycles. The discharge curves are very stable after 1800 cycles with no capacity fade indicating very minimal decay in energy density. **c,** Capacity versus cycle number for ~19wt.% MnO_2 loading electrode ran at 10C is shown. The results are similar to that of the cell shown in Figure 1c where there is no capacity loss and a ~100% coulombic efficiency is achieved. The high rate feature of the Cu^{2+} intercalated Bi-birnessite cathode broadens the scope of its utility in different applications. Inset shows the discharge curves for various cycles. **d,** Capacity(mAh/g) vs cycle number for the Cu^{2+} intercalated Bi-birnessite electrode is shown. The cell is cycled at 40C charge and **d**ischarge rate. The voltage-time curves for the different cycles is shown in the inset. The discharge characteristics of MnO_2 are still present after 6000 cycles.

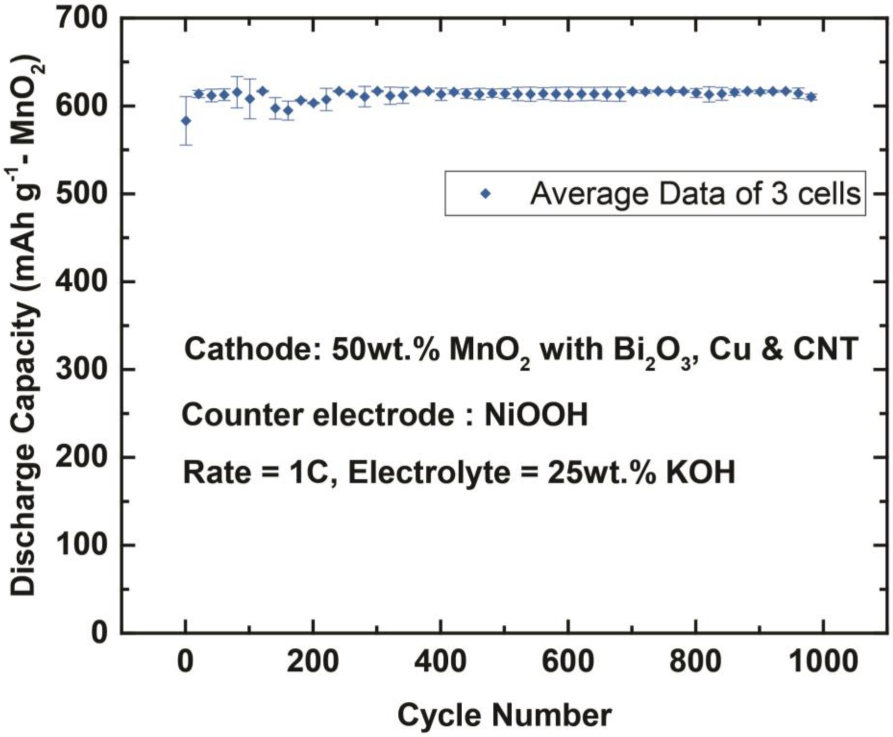

Supplementary Figure 3| Average data of discharge capacity vs cycle number of 3 similar cells showing statistical reliability of the data. Error bars show the standard deviation obtained from three similar cells cycled separately.

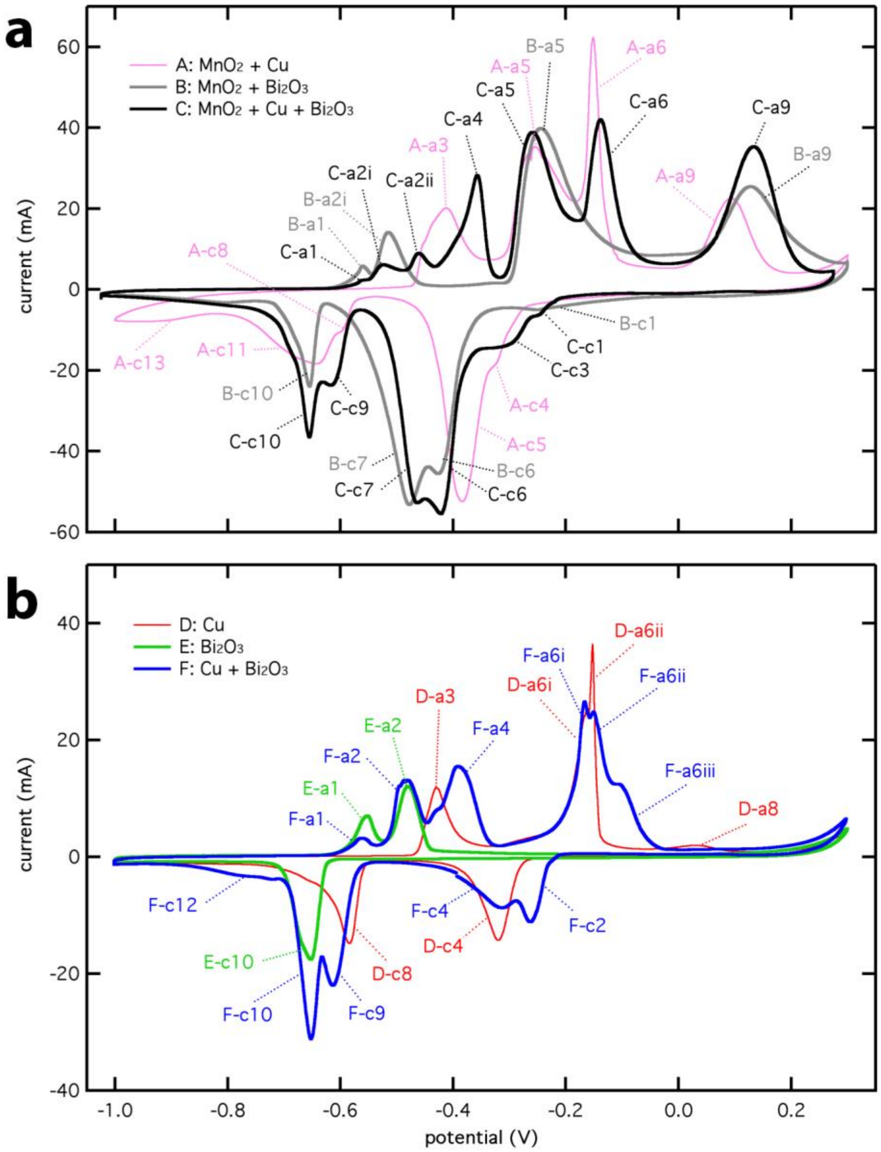

Supplementary Figure 4| Combinatorial cyclic voltammetry curves of individual components and addition of each additive to the MnO_2. a, Combinatorial cyclic voltammetry (CV) of six composite electrodes comprising graphite, MnO_2, and: Cu = A; Bi_2O_3 = B; Bi_2O_3 and Cu = C. **b,** Combinatorial CVs with graphite and: Cu = D; Bi_2O_3 = E; Bi_2O_3 and Cu = F. For all CV scans, cycle 5 is shown and represents a steady-state following electrode formation.

Supplementary Table 1|Combinatorial cyclic voltammetry of the composite electrodes (5th scan)

Peak [a]	Onset (V)<br>(vs. Hg|HgO)	Peak (V)<br>(vs. Hg|HgO)	Capacity (C)	Reaction [b]	Reference	Reference (V) [c]<br>Peak/Onset/Eq.
A-a3 [e]	-0.464	-0.412	16.74	$Cu^{0} \rightarrow Cu^{I}$		
A-a5 [e]	-0.307	-0.253	34.92	{Cu} $Mn^{II} \rightarrow$ {Cu} Mn^{III}		
A-a6	-0.174	-0.151	24.18	{Mn} $Cu^{0} \rightarrow$ {Mn} Cu^{II}		
A-a9	0.013	0.093	28.33	$Mn^{III} \rightarrow Mn^{IV}$		
A-c4	-0.257	-0.328	-8.76	$Cu^{II} \rightarrow Cu^{0}$		
A-c5	-0.323	-0.384	-39.25	$Mn^{IV} \rightarrow Mn^{III}$<br>$Mn^{III} \rightarrow Mn^{II}$		
A-c8	-0.570	-0.600	[d]	{Mn} $Cu^{I} \rightarrow$ {Mn} Cu^{0}		
A-c11 [e]	-	-0.642	-28.20	$[Cu^{I}\text{-}Mn^{III}] \rightarrow [Cu^{I}\text{-}Mn^{II}]$<br>$[Cu^{I}\text{-}Mn^{II}] \rightarrow [Cu^{0}\text{-}Mn^{II}]$		
A-c13	-0.806	-0.948	-12.47	{Mn} $Cu^{I} \rightarrow$ {Mn} Cu^{0}		
B-a1	-0.613	-0.561	2.17	$Bi^{0} \rightarrow Bi^{III}$		
B-a2i	-0.551	-0.515	7.14	$[Bi^{0}\text{-}Mn^{II}] \rightarrow [Bi^{III}\text{-}Mn^{II}]$		
B-a5	-0.292	-0.245	49.62	$Mn^{II} \rightarrow Mn^{III}$	Bode [f]	-0.066(p)
B-a9	0.013	0.128	39.49	$Mn^{III} \rightarrow Mn^{IV}$	Bode	0.252(p)
B-c1	-0.085	-0.248	-12.93	$Mn^{IV} \rightarrow Mn^{III}$	Bode	-0.066(p)
B-c6<br>B-c7	-0.375<br>-	-0.426<br>-0.478	-26.95<br>-44.69	$Mn^{IV} \rightarrow Mn^{III}$<br>$Mn^{III} \rightarrow Mn^{II}$	Bode	-0.335(p)<br>-0.404(p)
B-c10	-0.633	-0.655	-11.48	$[Bi^{III}\text{-}Mn^{III}] \rightarrow [Bi^{III}\text{-}Mn^{II}]$<br>$[Bi^{III}\text{-}Mn^{II}] \rightarrow [Bi^{0}\text{-}Mn^{II}]$	Bode	-0.669(p)
C-a1	-0.616	-0.559	[d]	$Bi^{0} \rightarrow Bi^{III}$		
C-a2i	-0.553	-0.523	4.13	$[Bi^{0}\text{-}Mn^{II}] \rightarrow [Bi^{III}\text{-}Mn^{II}]$		
C-a2ii	-0.493	-0.461	4.24	{Cu} $[Bi^{0}\text{-}Mn^{II}] \rightarrow$ {Cu} $[Bi^{III}\text{-}Mn^{II}]$		
C-a4	-0.404	-0.357	15.29	{Bi, Mn} $Cu^{0} \rightarrow$ {Bi, Mn} Cu^{I}		
C-a5	-0.301	-0.260	29.73	$Mn^{II} \rightarrow Mn^{III}$		
C-a6	-0.183	-0.138	26.09	{Bi, Mn} $Cu^{0} \rightarrow$ {Bi, Mn} Cu^{II}		
C-a9	0.037	0.133	46.98	$Mn^{III} \rightarrow Mn^{IV}$		

C-c1	-0.202	-0.250	[d]	$Mn^{IV} \rightarrow Mn^{III}$		
C-c3	-0.239	-0.310	-15.52	{Bi, Cu} $Mn^{IV} \rightarrow$ {Bi, Cu} Mn^{III}<br>{Bi, Mn} $Cu^{II} \rightarrow$ {Bi, Mn} Cu^{I}<br>{Bi, Mn} $Cu^{II} \rightarrow$ {Bi, Mn} Cu^{0}		
C-c6<br>C-c7	-0.375<br>-	-0.423<br>-0.463	-32.72<br>-29.63	$Mn^{IV} \rightarrow Mn^{III}$<br>$Mn^{III} \rightarrow Mn^{II}$		
C-c9	-0.574	-0.616	-9.99	{Bi, Mn} $Cu^{I} \rightarrow$ {Bi, Mn} Cu^{0}		
C-c10	-0.617	-0.656	-18.66	$[Bi^{III}\text{-}Mn^{III}] \rightarrow [Bi^{III}\text{-}Mn^{II}]$<br>$[Bi^{III}\text{-}Mn^{II}] \rightarrow [Bi^{0}\text{-}Mn^{II}]$<br>$[Cu^{I}\text{-}Mn^{III}] \rightarrow [Cu^{I}\text{-}Mn^{II}]$<br>$[Cu^{I}\text{-}Mn^{II}] \rightarrow [Cu^{0}\text{-}Mn^{II}]$		
D-a3	-0.466	-0.430	6.25	$Cu^{0} \rightarrow Cu^{I}$	He	-0.402(p)
D-a6i<br>D-a6ii	-0.211<br>-	-0.163<br>-0.153	15.45	$Cu^{0} \rightarrow Cu^{II}$	He	-0.136(p)
D-a8	-	0.032	<1	$Cu^{I} \rightarrow Cu^{II}$		
D-c4	-0.280	-0.319	-9.63	$Cu^{II} \rightarrow Cu^{0}$	He	-0.314(o) [g]
D-c8	-0.558	-0.583	-10.71	$Cu^{I} \rightarrow Cu^{0}$	He	-0.679(p)
E-a1	-0.621	-0.553	2.84	$Bi^{0} \rightarrow Bi^{III}$	Espinosa	-0.618(o)
E-a2	-0.522	-0.480	5.94	$Bi^{0} \rightarrow Bi^{III}$	Espinosa	-0.510(o)
E-c10	-0.626	-0.654	-8.78	$Bi^{III} \rightarrow Bi^{0}$	Bode<br>Gallaway<br>Vivier<br>Espinosa	-0.67(p), -0.8(p)<br>-0.65(o)<br>-0.62(p), -0.7(p)<br>-0.551(o)
F-a1	-0.625	-0.561	1.56	$Bi^{0} \rightarrow Bi^{III}$		
F-a2	-0.517	-0.483	7.34	$Bi^{0} \rightarrow Bi^{III}$		
F-a3	-0.467	-0.429	[d]	$Cu^{0} \rightarrow Cu^{I}$		
F-a4	-0.449	-0.391	11.13	{Bi} $Cu^{0} \rightarrow$ {Bi} Cu^{I}		
F-a6i<br>F-a6ii	-0.211<br>-	-0.167<br>-0.150	19.17	{Bi} $Cu^{0} \rightarrow$ {Bi} Cu^{II}		
F-a6iii	-	-0.105	[d]	{Bi} $Cu^{0} \rightarrow$ {Bi} Cu^{II}	He	~0.1(p)
F-c2	-0.229	-0.264 [b]	-5.92	{Bi} $Cu^{II} \rightarrow$ {Bi} Cu^{I}	He	-0.254(eq)
F-c4	-	-0.312	-7.00	$Cu^{II} \rightarrow Cu^{0}$		

F-c9	-0.574	-0.613	-8.78	{Bi} $Cu^{I} \rightarrow$ {Bi} Cu^{0}	Espinosa	-0.533(o)
F-c10	-0.619	-0.652	-11.14	$Bi^{III} \rightarrow Bi^{0}$		
F-c12	-	-0.767	~8	{Bi} $Cu^{I} \rightarrow$ {Bi} Cu^{0}		

a – As labeled in Fig. 3 and Fig. S7.
b – [Brackets] indicate known polynuclear complexes. {Braces} indicate the possible influence of a species, either through complexing, a substrate effect, or other causes.
c – Adjusted to Hg|HgO from the source reference. Key: (p) CV peak potential; (o) CV onset potential; (eq) equilibrium potential.
d – Peak capacity included with an accompanying peak.
e – Shows visible evidence of involving more than one faradaic reaction, e.g. peak doubling.
f – We attribute the somewhat large potential differences with the work of Bode et al. to conversion from an NHE reference.
g – In Figure 4 of the work by He, the reduction of Cu^{II} was observed at a potential of approximately -0.2 V vs. Hg|HgO. In our work, this potential was significantly lower, assigned to D-c4. As the electrode in our case was copper in the metallic state compressed with carbon, and in He's case it was planar polycrystalline copper, this can be explained by the variation in experimental conditions. The difference of ~80-119 mV implies a reduction to Cu^{0} as opposed to Cu_2O, as the potential is closest to the $Cu(OH)_2$/Cu couple of -0.314 V.

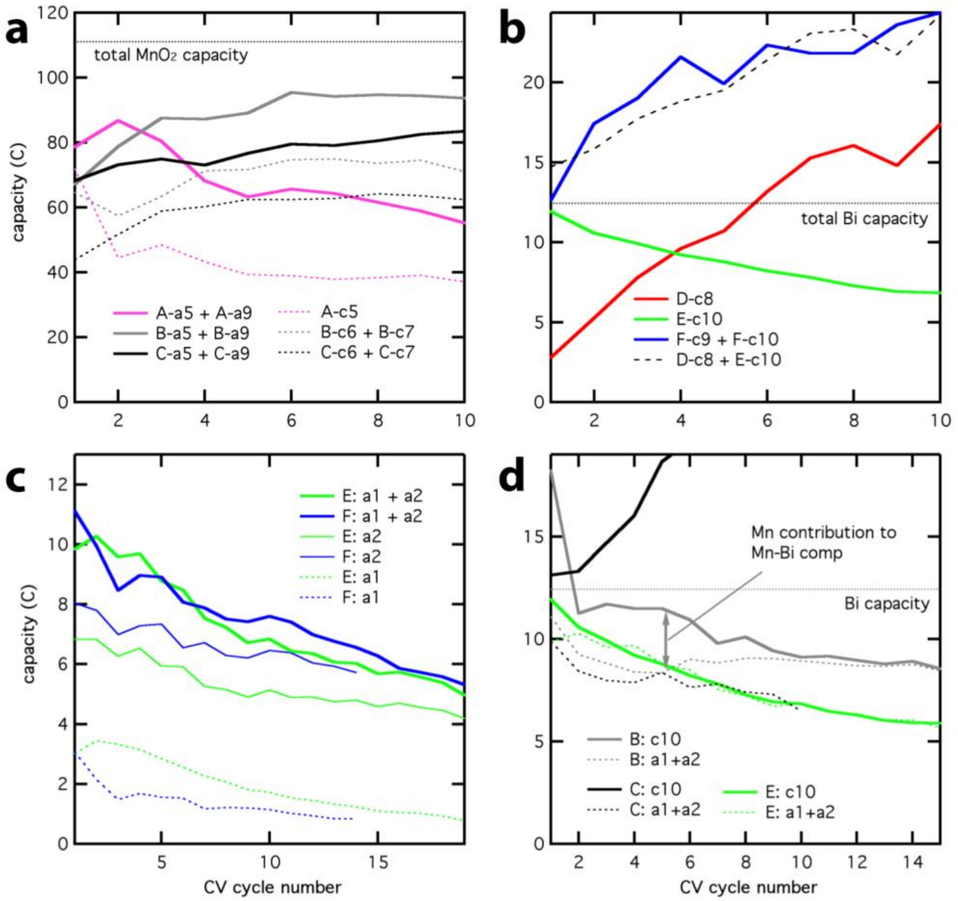

Supplementary Figure 5| Capacity analysis of the CV curves in Figure 4. a, Demonstration that the anodic Mn reactions have more capacity than the corresponding cathodic reactions in A, B, and C. This indicates Mn is reduced in the 'post-discharge' reactions as an ionic species. The post-discharge reactions are for the various cases (A-11 + A-13), B-10, and (C-9 + C-10). **b,** Demonstration that the 'post discharge' reactions in electrodes D, E, and F were additive, i.e. addition of the capacities in D and E were equal to that in F. **c,** Demonstration that the Bi oxidation reactions in electrodes E and F had equal capacity. Bi capacity deactivated during CV experiments with a half-life of 19 cycles, also shown in panels B and D. While the potentials of a1 and a2 in electrodes E and F were the same, the capacity of a2 was higher in electrode F. **d,** Demonstration that the Bi oxidation reactions have equal capacity in electrodes B, C, and E. These oxidations balance with Bi reduction in electrode E, but the post-discharge reactions in B and C have far more capacity. This indicates the existence of [Bi-Mn] compounds in B. In C the situation is more complicated as Cu reduction can also occur.

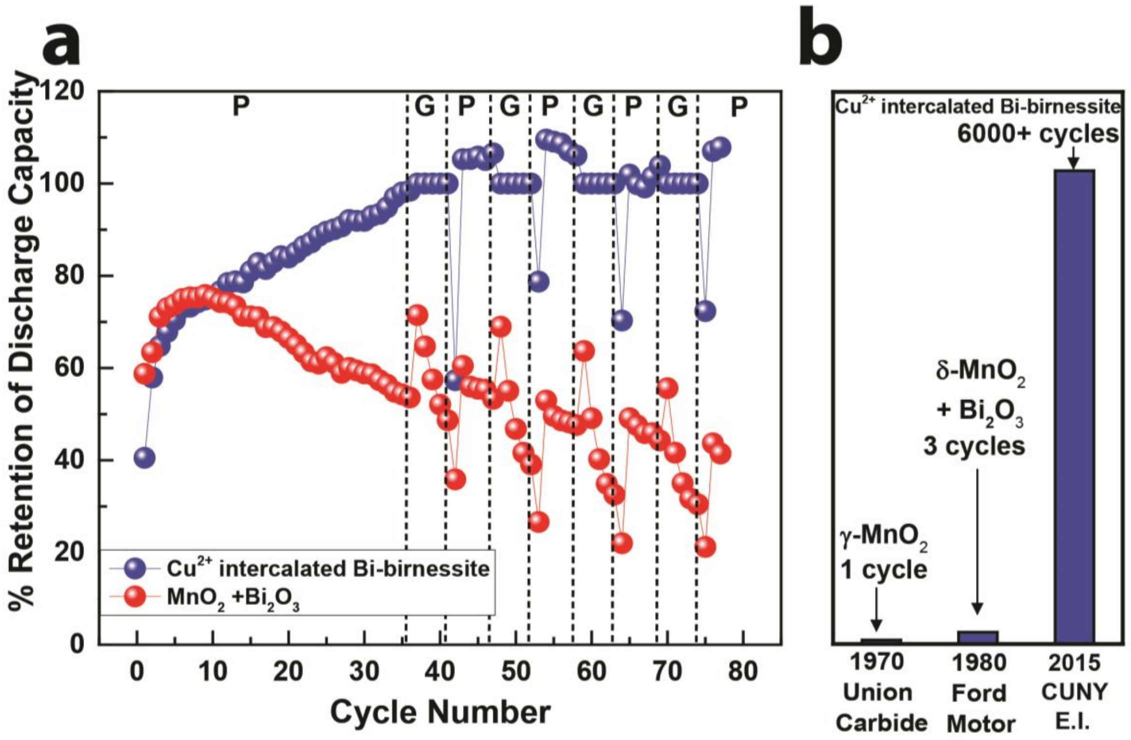

Supplementary Figure 6| Retention capacities of Cu^{2+} intercalated Bi-birnessite and MnO_2+Bi_2O_3 (Bi-birnessite) from potentiodynamic and galvanostatic curves. a, Retention capacity of MnO_2 electrodes with only Bi_2O_3 and of Cu^{2+} intercalated Bi-birnessite at different cycling protocols is shown. (G-Galvanostatic (at 1C), P-Potentiodynamic cycling protocols). **b,** Cycle life comparison of the best manganese dioxides cathodes in literature.

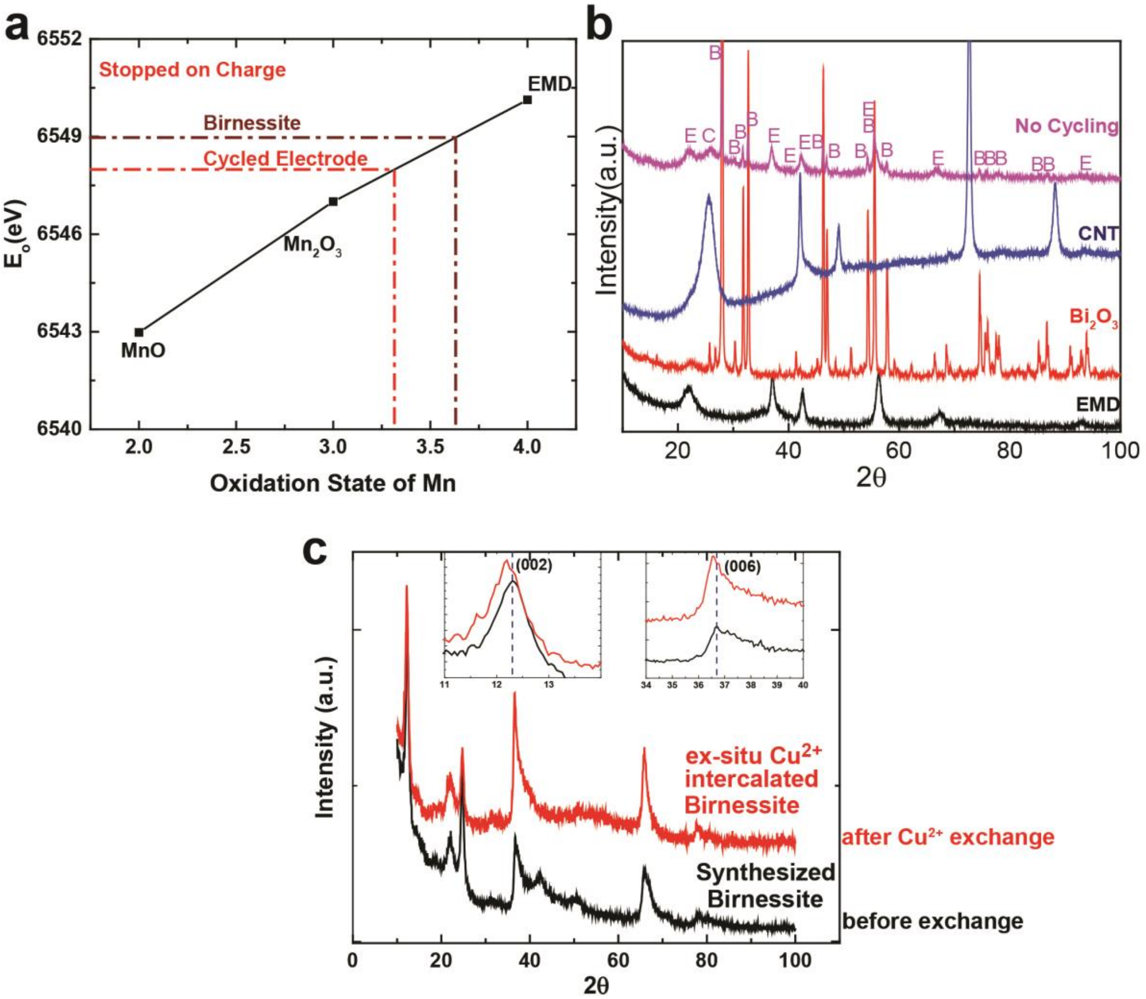

Supplementary Figure 7| Oxidation calibration curve and XRD patterns for confirming the presence of Cu^{2+}. a, Oxidation state calibration curve for manganese oxides K-edge. The cycled electrode has a lower oxidation state compared to the birnessite standard showing that there is presence of Cu^{2+} within the layers. **b,** XRD spectrums are shown for the individual components and mixture that comprised the No Cycling test case. E – EMD, B - Bi_2O_3 and C – CNT. **c,** XRD patterns for the synthesized and ex-situ Cu^{2+} intercalated birnessites.

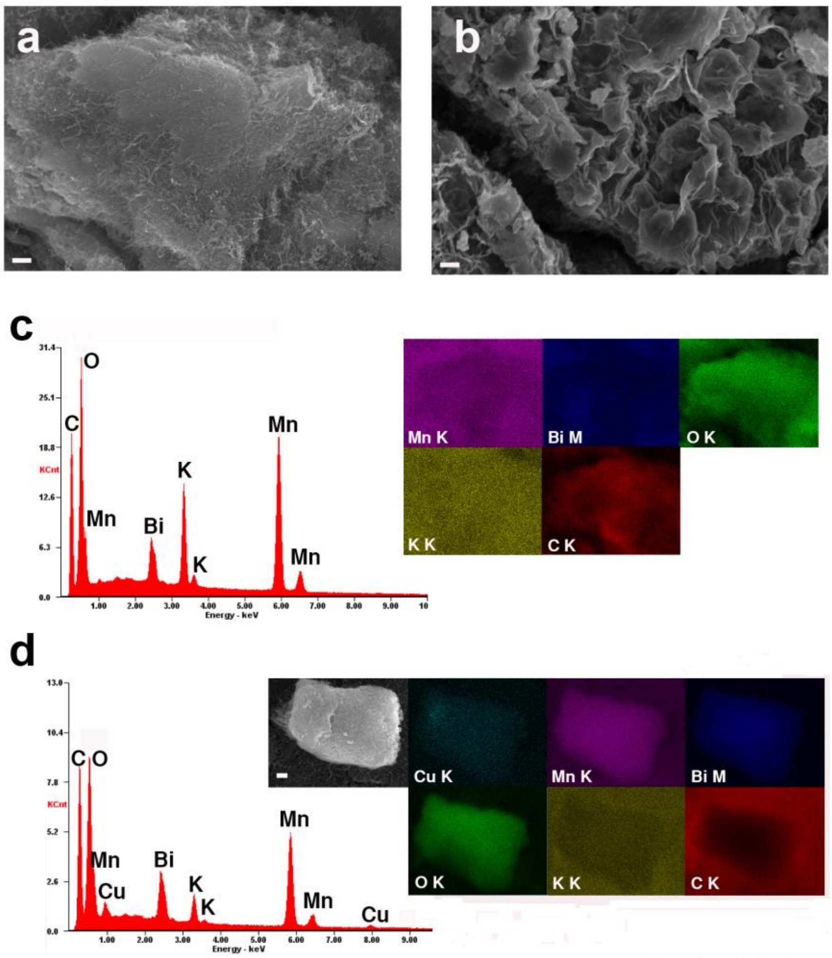

Supplementary Figure 8| SEM images and EDX mapping of the control and charged cycled electrodes. a, SEM image of the control electrode material is shown. The scale bar shown is 300nm. **b,** SEM image of the cycled electrode material is shown. The scale bar shown is 2μm. **c,** EDX spectra and x-ray mapping of the control electrode material shown in a. The elements are uniformly dispersed across the mapped area. The Mn to O atomic ratio is ~0.68 which corresponds to electrochemical inactive phase of hausmannite (Mn_3O_4). **d,** EDX spectra and x-ray mapping of a particle from the cycled electrode is shown. The scale bar shown is 200nm. The atomic ratio of Mn:Cu:Bi:K:O for the particle is 0.43:0.03:0.06:0.04:1.

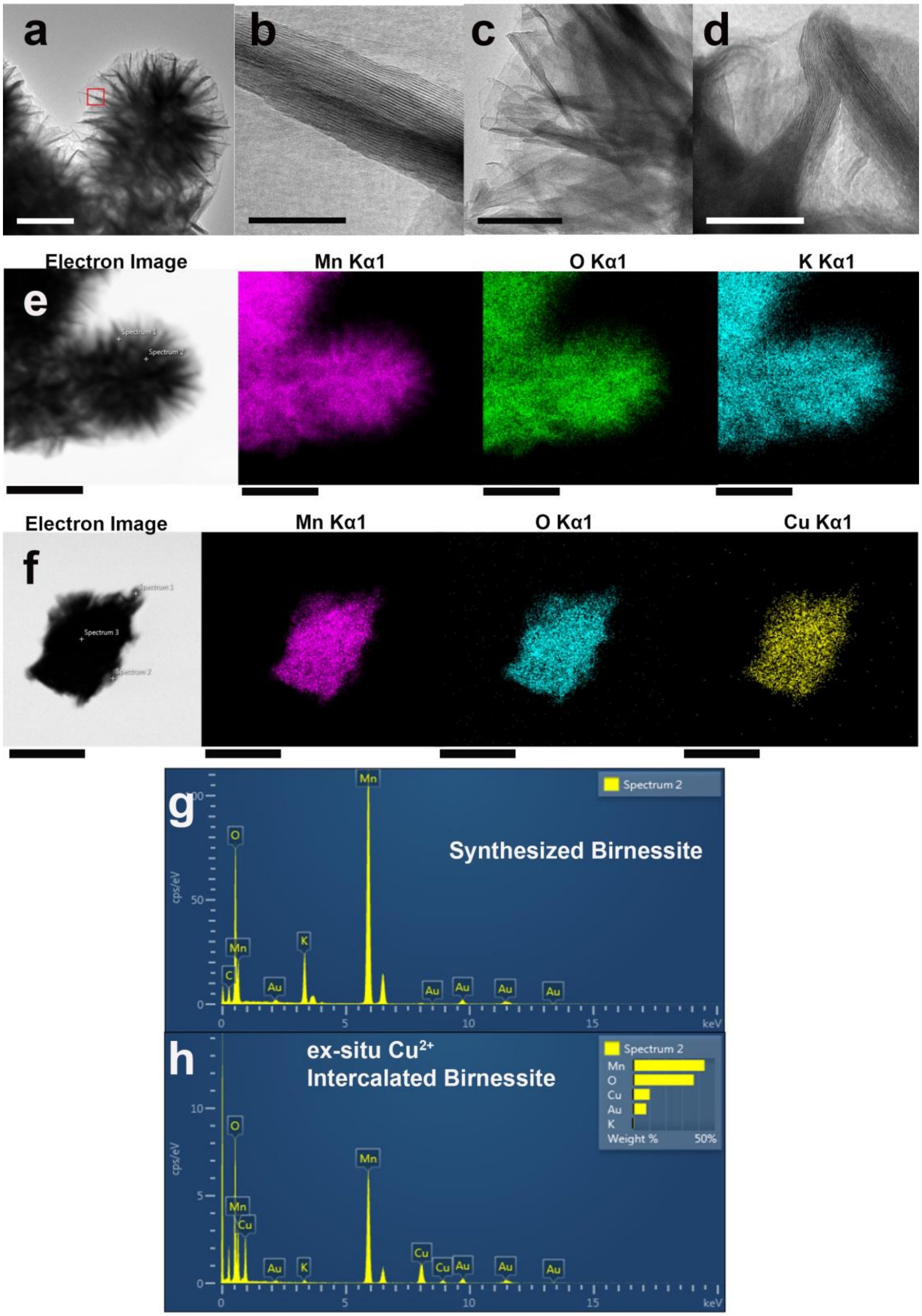

Supplementary Figure 9| TEM, HRTEM, EDX mapping and spectrum of the ex-situ synthesized birnessite and Cu^{2+} intercalation. a, TEM image of synthesized birnessite. **b,** HRTEM image of the red box shown in **a**. **c,** TEM image of the ex-situ Cu^{2+} intercalated birnessite. **d,** HRTEM image of the ex-situ Cu^{2+} intercalated birnessite. **e,** STEM image

and mapping of the birnessite particles shown in **a** & **b**. **f,** STEM image and mapping of the Cu intercalated birnessite particle shown in **c** & **d**. **g,** EDX spectrum of the particle shown in **a**.The gold peaks are from the TEM grid. The potassium peaks are from the reactants used in the synthesis procedure. The K atoms are usually situated in the interlayer spacings of the birnessite sheets. **h,** EDX spectrum of the particle shown in **c**. The gold peaks are from the TEM grid. The Cu completely replaces the K from the interlayer of the birnessite sheets. Scale bars are 500nm in (**a, e, f**), 10nm in **c** and 2nm in (**b,d**).

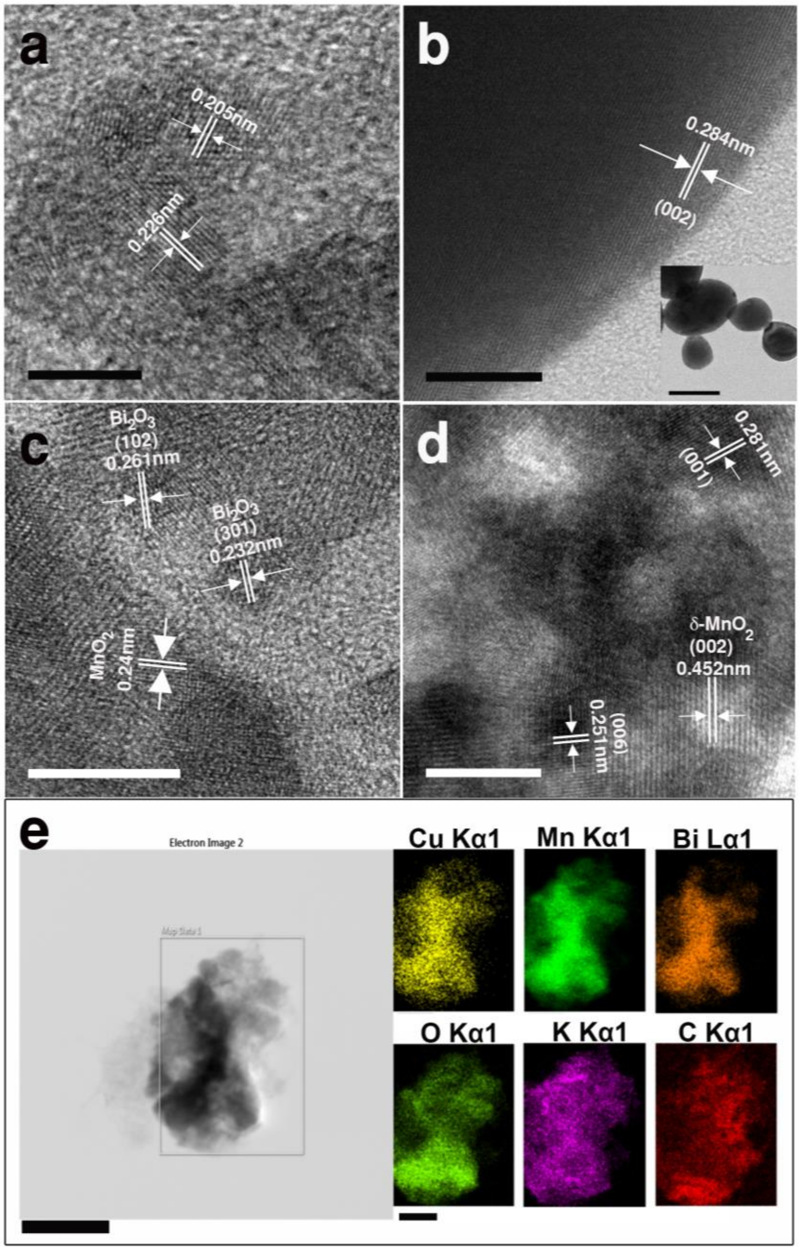

Supplementary Figure 10| TEM, HRTEM and EDX mapping of EMD, Bi_2O_3, 'no cycling' and cycled electrodes at charged state. **a,** HRTEM image of EMD is shown. The 0.205 nm lattice spacing is indexed to XRD 2-theta of 40.47^o, and 0.226 nm is indexed to 42.58^o. **b,** HRTEM image of Bi_2O_3 is shown. Inset is an image overview of Bi_2O_3 particles. The lattice spacing of 0.284 nm corresponds to the (002) direction. **c,** HRTEM image of the mix electrode material. The lattice spacing of 0.24 nm for EMD is indexed to 37.04^o, and the lattice spacing of 0.232 nm and 0.261 nm for Bi_2O_3 is indexed to (301) and (102) directions, respectively. **d,** HRTEM image of the cycled electrode material is shown. δ-MnO_2 lattices can be seen, where (002) and (006) directions are indexed. The (006) direction has a slightly higher d-spacing indicating intercalation of Cu^{2+} ions. **e,** X-ray mapping of the cycled electrode material is shown. The elements are evenly distributed in

the mapped region with the presence of Cu co-existing with that of Mn. Scale bars are 5nm in a, 10nm in (b-d), 100nm in b inset, 1μm in the electron image in e and 500nm in the elemental maps in e.

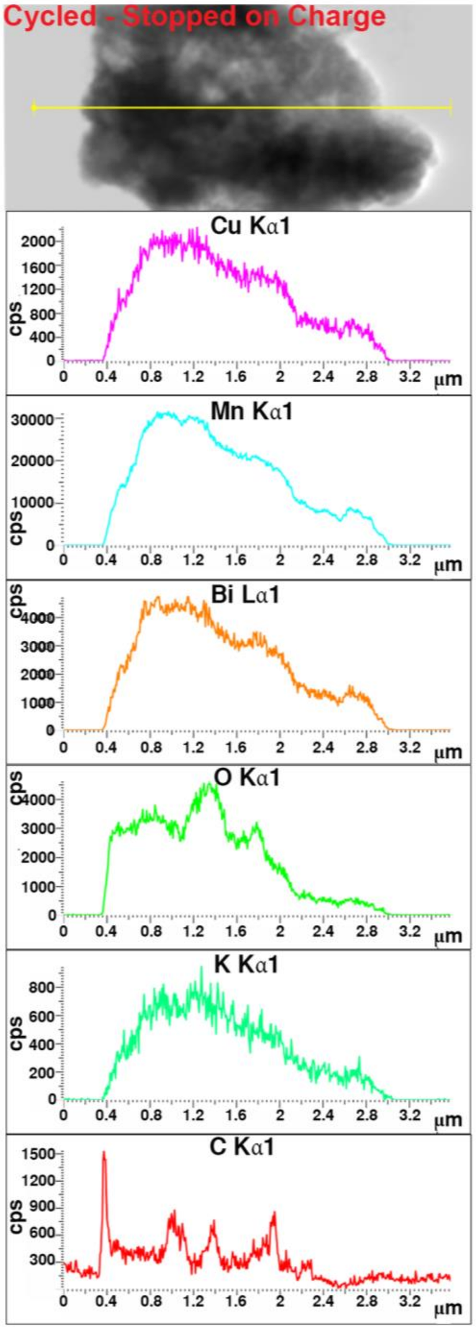

Supplementary Figure 11| Line scan TEM mapping of charged cycled electrodes. Line scan TEM mapping of cycled electrode material is shown, where the elements are evenly distributed in similar regions with the Mn counts being the highest. The presence of Cu in the same region of Mn indicates Cu2+ intercalation within the interlayers of the cycled electrode.

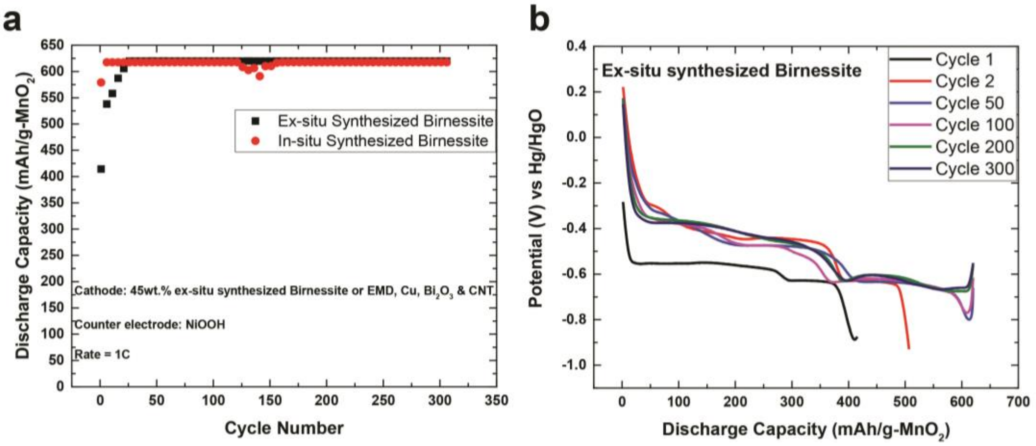

Supplementary Figure 12| Comparison of in-situ synthesized birnessite and ex-situ birnessite. a, Discharge capacity vs cycle number is shown for the ex-situ and in-situ synthesized birnessite. In the ex-situ synthesized birnessite, the mole ratio of the various elements was kept the same as the mole ratio in the in-situ formation of Bi-birnessite. There is no difference seen between the in-situ and ex-situ synthesized materials. **b,**Discharge curves of the ex-situ synthesized birnessite is shown, where the cycling curves are still stable after 300 cycles.

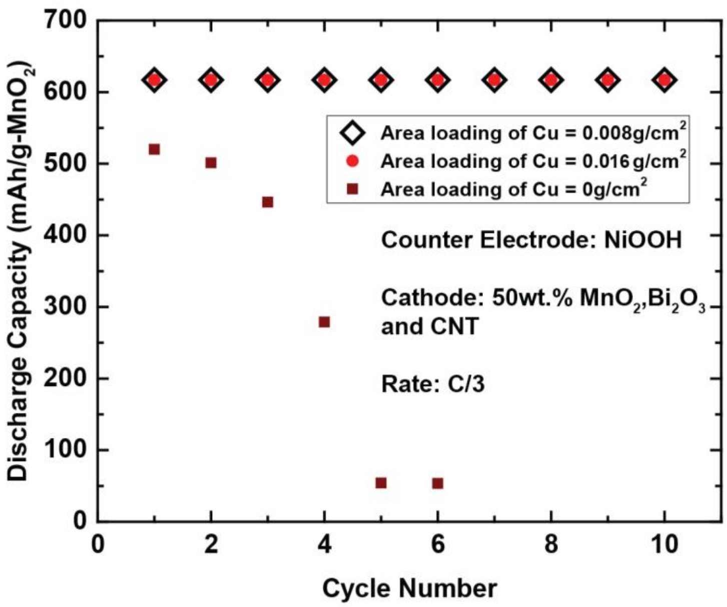

Supplementary Figure 13| Comparison of Birnessite performance with different Cu loadings. Areal Capacity is ~24mAh/cm^2.

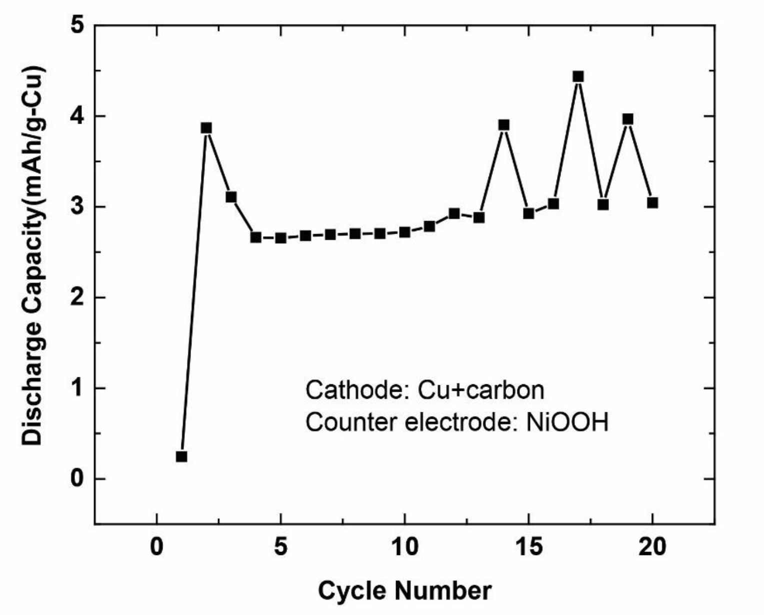

Supplementary Figure 14| Cycled capacity of a control electrode containing only copper and carbon. Rate = C/5.

Supplementary Discussion

An electroanalytical analysis provided evidence of both [Bi-Mn] and [Cu-Mn] compounds that participate in the cycling reactions of Cu^{2+} intercalated Bi-birnessite. Combinatorial cyclic voltammograms (CVs) in Supplementary Figure 4 identified the interactions of the cathode materials by methodically adding components to MnO_2 and then slowly sweeping electrode potential to observe the resulting faradaic reactions. (Full peak assignments are in Supplementary Table 1.) The recharging oxidations a5 and a9, in which $Mn(OH)_2$ was oxidized to δ-MnO_2, maintained the same character when Cu plus MnO_2, Bi_2O_3 plus MnO_2, or both Cu and Bi_2O_3 plus MnO_2 were present (i.e. compare oxidations A-a5, B-a5, and C-a5). The δ-MnO_2 reduction reactions, which represent discharge, were altered by additive formulation: any presence of Bi species provoked reactions c6 and c7, while the single reaction c5 occurred with Cu alone. The oxidation-reduction balance of each of these electrodes (Supplementary Figure 5) indicated Mn-species, which were reduced at potentials below those of c5, c6, and c7. As shown in Supplementary Figure 4b, both Cu and Bi, displayed reduction reactions in this range: D-c8 for Cu and E-c10 for Bi. In the MnO_2-containing electrodes, this meant MnO_2 capacity was reduced in the 'post-discharge reactions' (A-11 + A-13), B-10, and (C-9 + C-10). Bode and co-workers have previously reported the Bi-Mn compound using a similar rationale. For electrodes A and C, this 'post-discharge' reduction could occur as high as -0.58 V, while for B this could not occur above -0.64 V.

The interaction of Bi_2O_3 and Cu in the absence of MnO_2 was chiefly additive (Supplementary Figure 5b & c) except for the Cu reduction F-c2. The anodic reactions a1-a4 in electrodes A, B, and C were the additive species oxidizing out of the discharged material. Bi oxidation a1 always occurred at the same potential, but Mn shifted the potential of Bi oxidation a2 down to the a2i potential in electrodes B and C. Such a shift is consistent with a ligand increasing electron density on the Bi atom. In electrode C a second reaction a2ii not seen in any other case showed that Cu had an impact on Bi electrochemistry. The a1 and a2 reactions in all electrodes corresponded to the same coulombic capacity (Supplementary Figure 5d) proving they are Bi reactions.

Capacity of electrode A, containing MnO_2 and Cu, showed considerable fade by CV cycle 5 (Supplementary Figure 5a), but electrodes B and C showed stable capacity for ten CV cycles, shown in Supplementary Figure 3. After these initial ten cycles, electrode C increased capacity while electrode B decreased. As in the previous work of Yao, Bi_2O_3 alone as an additive showed rapid deactivation when subjected to galvanostatic cycling[21,22]. This effect was reversed by a potentiodynamic scan, although a long-term deactivation was still in evidence. The new cathode system in contrast was able to withstand both galvanostatic and potentiodynamic cycling.

Co-inclusion of Bi_2O_3 and Cu in a MnO_2 electrode increases electrode life, when cycled in any manner: potentiodynamically and/or galvanostatically. This is achieved by mitigation of Mn_3O_4 formation, which renders electrodes not containing Cu inactive in less than five galvanostatic cycles. As some Mn_3O_4 is observed in cycling electrodes, this must relegate its formation to non-critical locations that do not block electronic conductivity to the active material. Due to the high resistivity of Mn_3O_4, any electrode which contains an appreciable amount of it will be electrochemically inactive. Its spinel structure is built by an addition

reaction between the transient soluble Mn species $Mn^{III}(OH)_6^{3-}$ and $Mn^{II}(OH)_4^{2-}$. During discharge these species exist in the electrode pore space during reactions c5, c6, and c7, and this is when spinel-building occurs.

1 **Supplementary Table 2| Compilation of data from references used in plotting Figure 4c.**

REFERENCES	BEST CAPACITY (mAh/g)	CYCLE LIFE	RATES TESTED	CELL SIZE	AREAL LOADING (mg/cm^2)	AREAL CAPACITY (mAh/cm^2)	VOLUMETRIC CAPACITY (mAh/ml)	NOTES
**CUNY, 2016**	617	>6000	40C	2.54cm x 2.54cm	3.7	2.3	-	The cathode has 5wt.% MnO_2. The counter electrode is NiOOH.
**CUNY, 2016**	617	4500	20C	2.54cm x 2.54cm	19	~12	-	The cathode has ~19wt.% MnO_2. The counter electrode is NiOOH. This same areal capacity was also cycled at 10C as shown in Supplementary Figure 2.
**CUNY, 2016**	617	3500	1C	5.08cm x 7.62cm	33	~21	234	The cathode has 45wt.% MnO_2. The counter electrode is NiOOH.
**CUNY, 2016**	~360(chosen steady state value)	1750	4C	2.54cm x 2.54cm	52	~19	187	The cathode has 45wt.% MnO_2. The counter electrode is NiOOH.
**CUNY, 2016**	617	1000	1C	5.08cm x 7.62cm	39	~24	315	The cathode has 50wt.% MnO_2. The counter electrode is NiOOH.
**CUNY, 2016**	617(for 300 cycles) and ~500 after 1000 cycles	1000	C/3	5.08cm x 7.62cm	47	~29(for 300 cycles) and ~24(after 1000 cycles)	630(for 300 cycles) and 522(after 1000 cycles)	The cathode has 60wt.% MnO_2. The counter electrode is NiOOH.
**CUNY, 2016**	~600	90	C/3	5.08cm x 7.62cm	47	~28	140Wh/L against Zn anode	The cathode has 60wt.% MnO_2. The counter electrode is Zn. The Zn Depth of Discharge or utilization is 15%. A Zn utilization of ~35% will deliver >250Wh/L.
REF 16 & 17	521	20	C/20-1C	2.54cm x 2.54cm and 5.08cm x 7.62cm	33	18		Results of Prior Bi-Birnessite chemistry. CUNY repeated Wroblowa's experiments and found that under galvanostatic conditions the cathodes cycled poorly with high areal loadings. However, under potentiodynamic conditions, the electrodes replicated Wroblowa's results as shown in Supplementary Figure 6, which delivered 60-80% of the 617mAh/g.

REF 8,22,61	31	>3000	C/2-1C	5.08cm x 7.62cm	159	~5	~50	Karl Kordesch initiated the original idea of cycling a fraction of the MnO_2 theoretical capacity. CUNY repeated Karl Kordesch's tests on flat plate cells (5.08cm x 7.62cm) and were able to get 31mAh/g reversibly for >3000 cycles. CUNY demonstrated that even with shallow depth of discharge Zn-MnO_2 cells are feasible and energy dense for grid-scale applications. CUNY has also obtained ~62mAh/g over 3000 cycles.
REF 42	~450-500(for 200 cycles) and ~300-420(for 600 cycles)	200-600	C/2	N/R	N/R	N/R	N/R	Ref 41 showed the best galvanostatic cycling results of Bi-birnessite cathodes with high active wt.% loadings in literature. However, no areal loadings were reported. Cycled against NiOOH. The 200 cycles were obtained at 65% loading of their low surface area(LSA) cathodes, and the 600 cycles were obtained at 50% loading of their high surface area(HSA) cathodes. The LSA and HSA were synthesized and the detailed synthesis procedure is reported in their paper.
REF 40	~210	30	C/2 and C/4	N/R	Area not reported (5-8mg of active mass)	N/R	N/R	Cathode had 75wt.% EMD consisting of 0.5-5wt.% additives (Bi_2O_3, TiB_2 and/or TiS_2), 20wt.% Graphite and 5wt.% PTFE. Cycled against NiOOH in 9M KOH.
REF 41	~145	20	C/2	0.1cm pellet	N/R	N/R	N/R	Cathode consisted of a mix of EMD + Ag_3BiO_x. Ratio of Ag_3BiO_x to graphite to acetylene black was 5:2:1. Platinum foil was used as the auxiliary electrode.
REF 43	~150(C/2) and ~350(C/20)	50(C/2) and 13(C/20)	C/2 and C/20	N/R	N/R	N/R	N/R	50wt.% active loading. Zn was used as the counter electrode.
REF 44	~550(C/20) and ~345(C/4.2)	60	C/20 and C/4.2	N/R	~43	~26(for C/20)	N/R	Ref 45 used the same synthesis procedure as Ref 41 to synthesize their active material. Ref 45 synthesized the LSA material from Ref 41. However, Ref 45 mentions their areal loading. The anode they test against is a Zn sheet.

REF 62	~280(C/3)	5000	C/3 to 5C	CR2032 coin cells	1 to 5	1.4	N/R	70wt.% MnO_2 loading, 20wt.% carbon black and 10wt.% binder. α-MnO_2 was the polymorph of MnO_2 used. The electrolyte was acidic (2M $ZnSO_4$ with 0.1M $MnSO_4$). Zn foil was used as the anode. Weight of the Zn foil was not stated, therefore we cannot estimate the Zn DOD or utilization. A Zn foil usually has very low DOD or utilization (~ less than 1-2%). A practical energy dense battery usually has very high DOD on the Zn side, which is the cause of Zn failure.
REF 63	~225	30	20mA/g = ~C/11	0.11cm x 0.25cm area of mesh holes reported	Area not reported,12mg of active mass	N/R	N/R	

2

References

1. Bodé, M., Cachet, C., Bach, S., Pereira-Ramos, J.-P., Ginoux, J.C. & Yu, L.T. Rechargeability of MnO2 in KOH Media Produced by Decomposition of Dissolved KMnO4 and Bi (NO3) 3 Mixtures, I. Mn-Bi Complexes. *J Electrochem Soc* **144(3)**, 792-801 (1997).

2. Gallaway, J. W., Gaikwad, A. M., Hertzberg, B., Erdonmez, C. K., Chen-Wiegart, Y. K., Sviridov, L. A., Evans-Lutterodt, K., Wang, J., Banerjee, S. & Steingart, D. A. An in situ synchrotron study of zinc anode planarization by a bismuth additive. *J Electrochem Soc* **161(3)** A275-A284 (2014).

3. Vivier, V., Regis, A., Sagon, G., Nedelec, J.–Y., Yu, L. T. & Cachet-Vivier, C. Cyclic voltammetry study of bismuth oxide Bi2O3 powder by means of a cavity microelectrode coupled with Raman microspectrometry. *Electrochimica Acta* **46**, 907–914 (2001).

4. Espinosa, A. M., San José, M. T., Tascón, M. L., Vászquez, M. D. & Sánchez Batanero, P. Electrochemical behaviour of bismuth(V) and bismuth(III) compounds at a carbon paste electrode. Application to the study of the superconductor BiSrCaCuO, *Electrochimica Acta*, **36(10)**, 1561-1571 (1991).

5. He, J.-B., Lu, D.-Y. & Jin, G.-P. Potential dependence of cuprous/cupric duplex film growth on copper electrode in alkaline media. *Applied Surface Science* **253**, 689–697 (2006).
